# A workflow for modeling radiolysis in chemically, physically, and geometrically complex scenarios

**DOI:** 10.1016/j.isci.2025.112374

**Published:** 2025-04-08

**Authors:** Giuseppe De Salvo, Stefan Merkens, Andreas Körner, Birk Fritsch, Paolo Malgaretti, Andreas Hutzler, Andrey Chuvilin

**Affiliations:** 1 Electron Microscopy Laboratory, CIC nanoGUNE BRTA, Tolosa Hiribidea 76, 20018 Donostia-San Sebastián, Spain; 2 Helmholtz Institute Erlangen-Nürnberg for Renewable Energy (IET-2), Forschungszentrum Jülich GmbH, 91058 Erlangen, Germany; 3 Friedrich-Alexander-Universität Erlangen-Nürnberg, Department Chemical and Biological Engineering, Immerwahrstraße 2a, 91058 Erlangen, Germany; 4 Ikerbasque, Basque Foundation for Science, 48013 Bilbao, Spain

**Keywords:** Natural sciences, Chemistry, Physics

## Abstract

Radiation-based techniques contribute significantly to characterizing nanoscale samples across materials research but are frequently hampered by radiation-induced damage, particularly radiolysis in liquid media. The deep understanding and accurate modeling of radiation chemistry are crucial for interpreting experimental observations but are rarely sufficiently addressed in practice. We introduce a comprehensive workflow for numerically modeling radiolysis reaction kinetics in chemically, physically, and geometrically complex scenarios. The workflow streamlines the automatic composition of validated reaction networks from database files in a Python-based environment (AuRaCh tool) and their transfer to finite element computation environments (COMSOL Multiphysics software) for geometric and physical expansion. Its applicability is demonstrated in the context of liquid-phase electron microscopy but extends to other fields involving complex reaction networks. Model complexity is scrutinized, and potential simplifications are explored using characteristic numbers in experimentally relevant parameter regimes. The reported approach improves computational modeling and correlative experimental methods by promoting cross-community approaches.

## Highlights

• A workflow for modeling radiolysis in application-near scenarios is provided
• AuRaCh (chemistry) is streamlined with finite element methods (physics and geometry)
• Data for relevant radiolysis reaction networks (water and sparse gold chloride) is reported
• Model simplifications are discussed for applications beyond electron microscopy

## Graphical abstract

**Figure:**
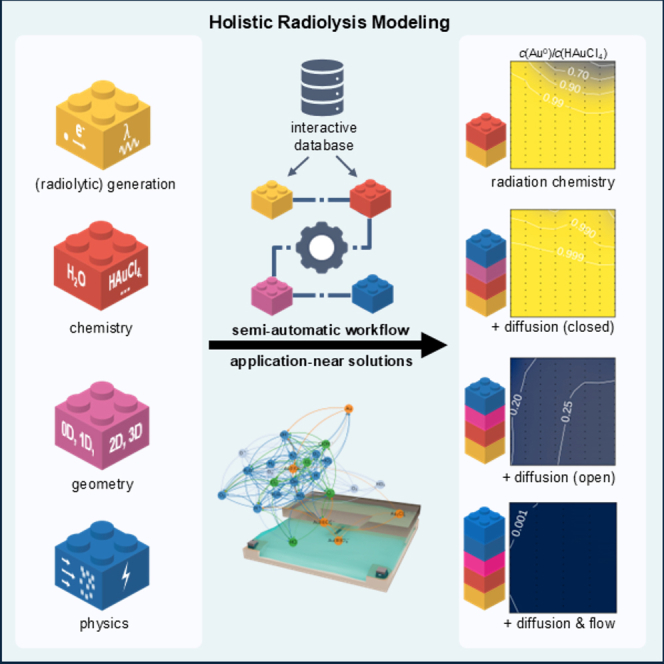

## Introduction

Radiation-based techniques are pivotal for sample characterization in material science.^1^^,^^2^ However, they confront a delicate dichotomy: shorter probe wavelengths yield more detailed information but increase the risk of damaging the sample due to the higher energy associated.^3^ This challenge particularly impacts fields that rely on ionizing radiation, such as high-energy electrons (e.g., electron microscopy) and photons (e.g., X-ray microscopy and spectroscopy) to monitor nanoscale processes.^4^^,^^5^^,^^6^

The most relevant damage mechanism in liquid media is radiolysis.^7^^,^^8^ It describes the scission of molecules generating a set of molecular, ionic, and radical species when exposed to ionizing radiation. Upon creation, these primary radiolytic species undergo chemical reactions (with other primary and newly produced secondary species) and diffuse, causing a complex reaction network to evolve in space and time.^8^ The time evolution of the reaction network strongly depends on the irradiation scenario and the composition of the irradiated sample, with continuous irradiation eventually leading to steady-state conditions. A strong foundation of radiation chemistry has been laid by combining experimental and theoretical approaches, which, among others, enabled a profound understanding of radiolytic reaction networks in homogeneous irradiation scenarios in the context of nuclear power plant engineering.^9^^,^^10^^,^^11^^,^^12^

The irradiation conditions of sample characterization methods in materials research, such as electron microscopy (in particular, liquid phase electron microscopy, LP-EM) and X-ray-based techniques (e.g., small-angle X-ray scattering, SAXS), differ considerably: Probe beams are typically focused on only a fraction of the liquid reservoir; that may vary over the time span of the irradiation, i.e., experiment, leaving significant portions of the chemically more complex reaction solution unirradiated.^13^ With respect to irradiation, electron microscopes (≈10^19^–10^26^ electrons m^−2^ s^−1^)^14^^,^^15^ and X-ray sources (≈10^15^–10^23^ photons m^−2^ s^−1^)^16^ operate at distinct probe flux densities. The resulting radiolytic damage depends on the energy absorbed by the sample. The absorbed dose rate is derived from the probe flux density by accounting for the sample’s (density-normalized) stopping power which varies with the nature and energy of the respective probe. Under typical conditions, the absorbed dose rates in electron microscopy (10^6^–10^13^ Gy s^−1^; where 1 Gy $\hat{=}$ 1 J kg^−1^)^17^ are considerably higher than those in X-ray-based techniques (10^0^–10^9^ Gy s^−1^)^18^ and nuclear power plants (10^0^–10^3^ Gy s^−1^),^19^ respectively. Probe focusing (e.g., in scanning transmission electron microscopy, STEM) generates local dose rates (up to 10^15^ Gy s^−1^) that surpass average values that apply to continuous irradiation scenarios (TEM mode).^20^

With respect to reservoirs, sophisticated model reactors have been developed that allow the sample environment to be controlled by cooling/heating, biasing, renewal, replacement, and/or mixing of the (confined) reaction solution.^21^ Their adaptation to respective requirements has led to various (customized) liquid cell (LC) architectures capable of replicating *ex situ* experimental scenarios.^22^ These aspects not only complicate the investigation of radiolysis in the context of material research, both experimentally and theoretically, but also call into question the legitimacy of a direct transfer of established knowledge from nuclear research to radiation-based characterization techniques. Revisiting the understanding of radiolysis is crucial to replicate relevant experimental conditions inside the model reactors quantitatively or to explain deviations thereof reliably.^23^

Radiolytic species have been associated with numerous phenomena, including gas bubble formation,^24^ phase transitions,^25^ growth^26^ and dissolution^15^^,^^27^ of nanoparticles, and solid-liquid interface effects,^28^ but their direct experimental quantification *in situ* remains challenging.^29^^,^^30^ Therefore, numerical modeling approaches comprise a significant portion of the work on radiolysis related to radiation-based characterization techniques.^25^^,^^31^^,^^32^ Numerous works have advanced computational methodology, particularly in the field of LP-EM. A comprehensive Python-based tool was introduced for the automated radiation chemistry simulation (AuRaCh) of complex reaction networks in homogeneous (also called zero-dimensional, “0D”) scenarios.^33^ This was complemented by finite element (FE) approaches, which have proven suitable to solve reaction networks in physically and geometrically more demanding scenarios with adjustable spatiotemporal resolution.^17^^,^^34^ State-of-the-art (FE) radiolysis models encompass simple water reaction sets^17^ complemented by chemically more comprehensive examples such as (sparse)^35^ gold,^33^^,^^36^ silver,^37^^,^^38^ iron,^39^^,^^40^^,^^41^ chlorine,^33^^,^^36^^,^^39^ and organic additive^37^^,^^42^^,^^43^ sets.

Regarding physics, diffusion is the most frequently accounted transport mechanism (usually in low-dimensional model geometries that exploit rotational symmetry).^17^^,^^36^^,^^44^ Only occasionally, diffusion was superimposed with convection in more complex model geometries.^34^ The efforts to increase model complexity are typically limited to either chemical *or* physical (in tandem with geometric) extensions, with only a few comprehensive attempts existing.^45^ Implementations further barely exceed continuous irradiation scenarios (i.e., TEM mode).^46^ Moreover, these models are complemented with, but often not coupled to, models addressing interface processes,^41^^,^^47^ solution mixing,^48^ electro-^49^ and surface chemistry.^50^ While radiolysis simulations are typically attempted to be correlated with experimental observations,^37^ insufficient replication of the physical, chemical, and geometric complexity can mislead interpretations if the computed scenarios lack accuracy. However, the degree of validity of model simplifications and the accuracy required remains unclear. In addition, the methodological implementation of application-near, thoroughly validated radiolysis models in correlative workflows remains vacant.^51^

Advances to increase the physical, chemical, or geometric complexity of radiation chemistry models are thwarted by an extensive list of challenges, which comprises (1) the difficulties of composing expanded reaction networks that require collecting chemical reaction pathways and corresponding parameters such as reaction rate constants, diffusion coefficients, and radiolytic generation values from literature or through experimentation; (2) the susceptibility for errors when implementing them manually; (3) the adequate replication of geometric and physical complexity; and (4) the substantial rise in computational costs when solving increasingly multidimensional problems.

Overcoming these limitations has the potential to achieve more realistic modeling of complex reaction kinetics for applications related to material research. The ideal radiation chemistry model would describe experimental scenarios sufficiently accurately to provide robust predictions with minimal computational costs and an appropriate number of degrees of freedom. Depending on the purpose of the model, this includes (1) the (electro)chemical reaction kinetics of the sample of interest, (2) their coupling to radiation chemistry, (3) considering the accurate spatiotemporal (continuous or scanning beam) irradiation together with (4) relevant mass transport mechanisms, i.e., diffusive and convective flux as well as drift in (externally applied or locally generated) electric fields, (5) in realistic reactor geometries, (6) considering beam-induced secondary irradiation (electrons and photons), (7) (liquid-solid-gas) interface transitions, (8) as a function of temperature and pressure.

Herein, we introduce a workflow for reliable radiation chemistry modeling in multidimensional liquid cell architectures and irradiation scenarios. The workflow integrates our previous approaches to modeling radiolysis in chemically^33^ as well as geometrically and physically^34^ challenging scenarios. It streamlines the assembly and validation of complex reaction networks in AuRaCh, their transfer into an FE modeling infrastructure (COMSOL Multiphysics software), and subsequent expansion in non-homogeneously irradiated geometries considering relevant mass transport mechanisms. Implementing a series of radiolysis reaction networks is demonstrated for different geometric configurations (with closed and open boundaries) corresponding to relevant LP-EM reactor setups accounting for diffusion and convection under continuous electron irradiation. The results are discussed alongside the impact of complex implementations on the accuracy achieved and the computational costs required. The need for advanced reaction kinetic modeling for radiation-based characterization techniques is scrutinized, and the potential for simplifications is reviewed based on characteristic numbers in experimentally relevant parameter regimes.

### Developing a comprehensive modeling workflow

#### Realistic radiation chemistry modeling

Numeric models that emulate radiolysis reaction kinetics in radiation-based sample characterization experiments contain two parts: the (radiation) chemical reaction network and its expansion into geometric models that consider spatiotemporal irradiation and coupled physics such as mass transport (i.e., diffusion, convection, and drift in an electric field). The differential equation that describes the concentration field of the species *c*_i_ = *c*_i_(*x,y,z,t*) is denoted in Equation 1 in a generalized form:

(Equation 1) $$\frac{dc_{i}}{dt}=\rho \Psi G_{i}+\sum_{j}k_{j}\left(\prod_{l}c_{l}\right)-\sum_{m\neq j}k_{m}\;\left(\prod_{n}c_{n}\right)+D_{i}\nabla^{2}c_{i}+v\nabla c_{i}-\nabla \left(z_{i}u_{i}c_{i}\nabla \varphi \right)$$

Of the right-hand terms of Equation 1, the first three describe the radiation chemistry network (radiolytic generation as well as chemical production and consumption). The fourth, fifth, and sixth terms describe mass transport due to diffusion, convection, and drift in an electric field. All variables, material constants, and their implications are defined in the next section.

#### Challenges in reliable model implementation

Realistic radiation chemistry modeling requires the precise implementation of the chemical, physical, and geometric complexity of the problem, i.e., the accurate definition of all parameters in Equation 1. Even though the complications are manifold, comprehensive similarities can be identified for various scenarios and reasonable simplifications can help simplify the real scenario.

#### Chemical challenges

The radiolytic generation rate (1^st^ right-hand term in Equation 1) of a species *i* is proportional to the irradiated solvent’s density (ρ, in kg m^−3^), the dose rate (*ψ,* in m^2^ s^−3^ = Gy s^−1^) and further described by specific generation values (*G*_i,_ in mol J^−1^; refer to the Supporting Information section 1A for further details).^17^ In principle, *G-*values depend on the type and characteristics of the applied radiation and the irradiated material. *G-*values were meticulously determined for aqueous solutions^52^ and are also available for some organic solvents, including various alcohols.^53^^,^^54^ In scenarios where exact *G*-values are unavailable, they must be determined from experimentation or approximated based on existing knowledge. In LP-EM, transferring *G*-values from pulse radiolysis studies and stochastic modeling is common practice.^52^ It is further a common simplification to neglect the radiolytic generation of species from solutes due to the much lower concentration than the solvent. This dilution approximation works well for solute concentrations smaller than 0.1–1 M.^55^^,^^56^ To convert an electron flux density *φ* (A m^−2^), a commonly measured quantity in electron microscopy, to the dose rate absorbed by the liquid layer, the following approximation holds (if *t* is comparable to $\lambda_{\text{IMFP}}$):^57^^,^^58^^,^^59^

(Equation 2) $$\psi =\frac{\varphi }{e}S\left(1+\frac{t}{\lambda_{\text{IMFP}}}\right)\text{.}$$

In Equation 2, *S* is the density-normalized stopping power (m^4^ s^−2^), *e* the elementary charge (A s^−1^) to convert *φ* to a number density, *t* the liquid layer thickness, and $\lambda_{\text{IMFP}}$ is the inelastic mean free path of the probe in water (both in m).

The concentration evolution of each species *i* in the network is described by the entirety of all reactions in which it is consumed (3^rd^ term of Equation 1) when reacting with present and produced reactants *j* or created (2^nd^ term) in their reaction with each other. The rate of each reaction is defined by the concentration *c* of the involved species (*c*_l_ and *c*_n_) and a rate constant (*k*_j_ and *k*_m_ – for production and consumption, respectively). *G*-values and rate constants depend on temperature.^60^ To achieve chemical accuracy, the radiolytic reaction subset of the solvent must be coupled to a subset that chemically describes the monitored sample. While solvent subsets (reaction equations alongside corresponding rate constants) are rather well tabulated (particularly) for water,^61^ sample-specific subsets occasionally require assembly from scratch. A sufficient network completeness for a given scope is crucial for reliable expansion, but its construction may test the resilience of the most obsessive scientists. Owing to the generality of reaction(constant)s, the required information may be found well-documented in related research fields,^62^ as are methods to determine them experimentally.^61^ Nevertheless, their faultless transfer from literature into a computational model presents a (time-intensive) challenge, given that it must be performed manually for hundreds of reaction (constant)s and each implementation individually. Recent work suggests that the individual precision of a network description is often only of interest with respect to their order of magnitude, as shown by sensitivity analysis for irradiated aqueous H_2_SO_4_ solutions.^29^ Sparsing a model can help to reduce the number of reactants and thus computational costs when solving Equation 1, while ensuring high precision.^35^ However, this requires comparison to a ground truth, which is not always known.

#### Physical and geometrical challenges

Spatially inhomogeneous irradiation scenarios and the application of external physics (e.g., fluid flow and/or electric biasing) further increase the model complexity, requiring a space-dependent implementation of Equation 1. The accurate implementation of physical aspects is closely connected to the accurate geometric replication of the experimental setup.

Beyond a limited number of use cases with homogeneous irradiation (e.g., in graphene liquid cells and derivatives),^63^ the probe beam irradiates only a fraction of the reactor volume. This requires considering diffusion. Diffusive transport is expressed through Fick’s second law (4^th^ term in Equation 1), where $\nabla^{2}c_{i}$ equals the (negative) divergence of the diffusive flux ($-\nabla J_{D}$) and *D*_i_ denotes the diffusion coefficient of each species *i*. Diffusion coefficients are known for most solutes (typically 10^−9^ – 10^−10^ m^2^ s^−1^), but non-classical phenomena such as slip-stick motion and Levy flights due to electrostatic interactions with the membrane were also reported.^64^^,^^65^

In flow scenarios, convective transport superimposes diffusion, usually unidirectionally perpendicular to the probe beam. Determining the flow velocity field inside a realistic flow channel geometry is challenging but imperative for detailed radiation chemistry modeling. Given the shortcomings of experimental approaches,^66^ flow field estimations rely on solving the Navier-Stokes equation for experimentally applied volumetric flow rates, *Q,* which requires an exact model replication of the channel geometry.^48^^,^^67^ If the overall flow rate (*Q*_total_) is time-invariant (as typically the case), *v* in Equation 1 acts as a space-dependent constant (*v = v*(*x,y,z*)) that can be determined in an independent computation step preceding the computation of the reaction kinetics leading to *c*_i_. The dimensions of the flow reactors, the solvents used, and the (total) flow rate applied typically imply laminar flow conditions characterized by parabolic flow profiles, as predicted by the Reynolds number.^48^^,^^68^

The presence of electric fields, e.g., in electrochemical experiments or due to beam-induced charging of insulating membranes, induces the drift of charged species, which must be considered for a complete description (6th term of Equation 1). Similarly, it affects rate constants at interfaces which enforce a static orientation.^69^^,^^70^ Drift is proportional to the charge number*, z*_i_, and mobility, *u*_i_, of each ionic species *i*, but foremost depends on the (local) electric field strength, which is expressed as the gradient of the potential (∇φ) in Equation 1. While *z*_i_ is trivial and *u*_i_ typically well-documented, the electric field strength is – analogously to the flow velocity field – to be computed on a geometrical replication of the electrode configuration, which turns out an even greater challenge with few examples reported.^49^^,^^71^ For most employed electrode configurations, highly complex electric fields occur, including the appearance of hotspots.^1^

#### Symmetry considerations

Symmetry operations may be applicable to reduce model complexity and, thus, computational costs; to avoid loss in model accuracy, the nature of acting mass transport mechanisms must be respected. For simple reaction-diffusion scenarios, the concentration depends solely on the radial distance from the beam (center). Hence, 1D models (with radial symmetry) can accurately capture these scenarios. However, when convective transport and/or drift are relevant, more complex 2D or 3D implementations are required. Considering the comparable geometry of irradiation/reactor configurations, similar simplifications may be valid for the different characterization techniques discussed but may further depend on the length scales (see below).

#### A holistic modeling workflow

**Figure 1:**
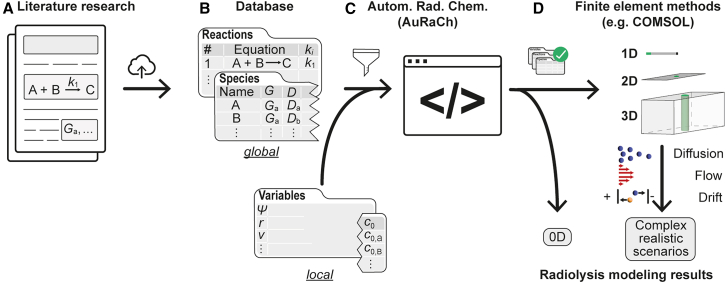
Illustration of a comprehensive radiolysis modeling workflow (A and B) Relevant parameters that define chemical reactions (reaction constant *k*_i_) and involved species (radiolytic generation value *G*_i_, diffusion coefficient *D*_i_) are collected from the research literature (A) and uploaded to a (global) database (B) once. The database is complemented with experiment-specific information describing the irradiated solution (e.g., initial concentrations *c*_0_), beam parameters (e.g., dose rate ψ and radius *r*) and additional physics/external stimuli (e.g., fluid flow velocity *v*). (C and D) A reaction set is composed from database tables, and its physical integrity is validated in AuRaCh (C),^33^ which already provides facile 0D reaction kinetic modeling,^33^ (D)). Finally, the validated reaction set is transferred into a user-friendly environment for finite element modeling that facilitates the simulation of realistic irradiation scenarios and precise consideration of all relevant mass transport mechanisms, i.e., diffusion, convection, and drift. This study demonstrates the transfer of validated 0D reaction sets by interfacing a MATLAB code with COMSOL Multiphysics software, enabling their expansion in complex spatiotemporal scenarios.

The diverse challenges encountered in realistic radiation chemistry modeling call for a versatile and automated modeling workflow, as shown in Figure 1. The suggested workflow is built upon combining input from local and global databases: (1) The *global* database (Figure 1B) contains all general information on chemical reactions together with the corresponding rate constants, *k*_i_, and details on the chemical species *i* involved such as their radiolytic generation values, *G*_i_ and diffusion coefficients, *D*_i_. We consider open access to such a database crucial to unifying modeling approaches and facilitating its ongoing expansion (predominantly through literature or experimental research; Figure 1A) by the community.^72^^,^^73^ (2) The *local* database contains experiment-specific information on the irradiation scenario (e.g., dose rate ψ and radius *r*), additional physics/applied stimuli (e.g., fluid flow velocity *v* and electric bias *V*) as well as the irradiated solution (e.g., initial concentrations *c*_0;_ Figure 1B). Based on the databases, the workflow semi-automatically composes verified reaction networks for each specific implementation. To this end, the global database is filtered for involved reactants based on the user’s request and the integrity of the constructed reaction set is verified with respect to charge and mass balance as well as sparsity (Figure 1C). Upon network simulation and analysis for homogeneous irradiation conditions, the validated reaction set is transferred into an environment for FE modeling that facilitates the computation of heterogeneous irradiation scenarios and precise consideration of all relevant mass transport mechanisms, i.e., diffusion, convection, and drift (Figure 1D).

### Workflow implementation

Combining global and local input files makes the proposed workflow versatile across various areas, comprising radiation-based characterization techniques such as LP-EM and X-ray-based methods. In this manuscript, we benchmark the workflow in the context of LP-EM.

#### Database configuration

A primitive *global* reaction database was composed to facilitate the demonstration of the proposed workflow using models from previous research as a reference. The global database files comprise (1) a *reaction library* table containing the chemical reactions and (2) a *species* table containing the radiolytic generation values and diffusion coefficients of the species contributing to the *reaction library.* Both files were assembled from previously constructed sub-sets.^17^^,^^33^^,^^37^^,^^39^^,^^40^^,^^45^^,^^74^ The *species* table was complemented with local experiment-specific information on the irradiated solution (initial concentrations) as well as (3) a *local variables* table containing beam parameters and flow velocities. The parameters were chosen to match the reference models to facilitate workflow validation.^17^^,^^35^ Refer to the STAR Methods section for details on database configuration and access to the files.

#### Network filtering, verification, transfer, and expansion

This manuscript centers on the filtering and validation of a reaction network in 0D and its transfer to geometrically and physically expanded models. Therefore, AuRaCh was equipped to filter the global database based on the user’s request and verify the integrity of the constructed reaction set with respect to charge and mass balance. In contrast, (manual) reaction network sparsing was introduced previously.^35^ To transfer the verified reaction network, COMSOL Multiphysics software was interfaced with MATLAB software, allowing the construction and expansion of models based on AuRaCh output files. AuRaCh and the corresponding MATLAB script (Rad-4D) are available open-source (see STAR Methods section). Practically, in COMSOL, a homogeneous reaction kinetic model was set up from the AuRaCh output files, which provided (1) the chemical reactions, (2) details on the involved species (i.e., diffusion coefficient *D*_i_, radiolytic generation values *G*_i_) as well as (3) experiment-specific information (i.e., dose rate ψ, beam radius *r,* liquid layer thickness *t* and average flow velocity $\bar{v}$). The functionality to generate space-dependent models in a FE platform (here: COMSOL Multiphysics) was then exploited to expand the reaction set in arbitrarily complex geometries. Finally, additional initial and boundary conditions were defined to account for the geometrical and physical peculiarities of different, relevant experimental scenarios (see STAR Methods section).

#### Symmetry considerations for common LP-EM scenarios

**Figure 2:**
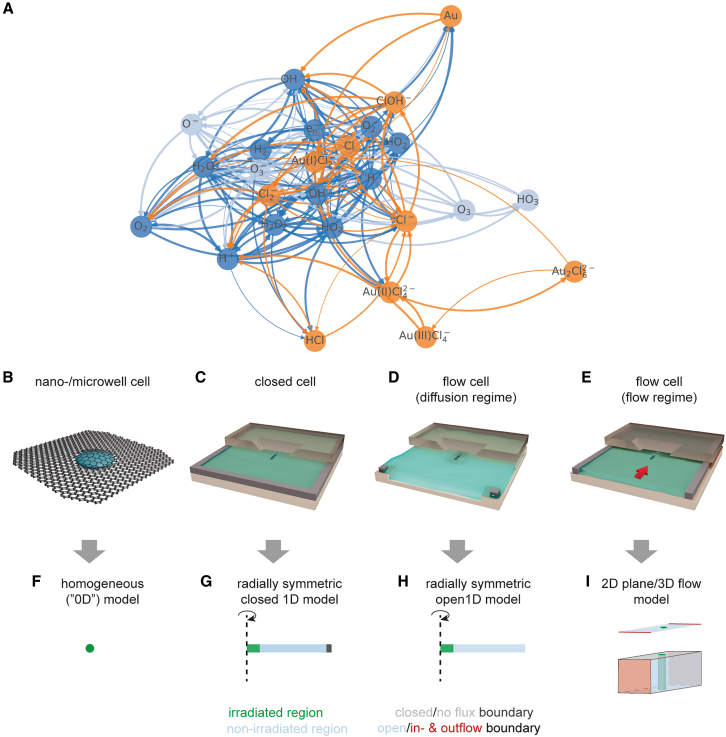
Radiolysis reaction network modeling in realistic LP-EM reactor geometries (A) Graph theoretical depiction of an assembled radiolytic reaction network. The composition of networks for increasingly realistic and effective chemical modeling by combining sub-sets (*dark-blue* and *orange*) and sparsing (*grey-blue*) is illustrated. Each node represents a chemical species, and edges (arrows connecting nodes) indicate the consumption/production of the species due to a chemical reaction. (B–I) The irradiation/reactor configuration in experiments (B–E) determines the geometric complexity required for realistic modeling (F–I): (B) closed cell configurations where the beam size is at least comparable to that of the liquid cell can be replicated in a homogeneous (“0D”) model, (F); (C) closed cell configurations where the beam size is significantly smaller than that of the liquid cell can be replicated in a 1D heterogeneous implementation (with closed boundary condition and radial symmetry, (G); (D) open cell configurations with only diffusion can be replicated in a 1D heterogeneous implementation (with open boundary and mostly radial symmetry, (H); and (E) open cell configurations with convection superimposed to diffusion require a 3D, or potentially 2D, implementation, (I). In this work, models are solved with respect to time, which further increases complexity and computational costs.

Implementing complex reaction networks (Figure 2A) in expanded scenarios comes with increased computational costs. Figures 2B–2I summarize the most relevant experimental LP-EM scenarios, and the following paragraphs report valid geometric simplifications based on fundamental symmetry considerations and addressed physics that does not reduce the accuracy of the model.

In closed cell configurations, the irradiated solution is isolated: If the probe beam size exceeds the liquid cell dimensions and ensures a homogeneous intensity profile over the cell, overall diffusive transport cancels out resulting in zero net flux. Under these conditions, a homogeneous (space-independent) model (“0D”, Figure 2F) represents a valid simplification. This applies, for instance, to graphene liquid cell (Figure 2B) and microwell/-cavity setup.^22^^,^^75^^,^^76^ Conversely, spatial concentration gradients arise if the beam does not uniformly irradiate the entire liquid cell volume. In such scenarios, a 1D heterogeneous model geometry with closed boundary conditions and, apart from a few exceptions,^77^ radial symmetry (Figure 2G) is required. Micro-electro-mechanical systems (MEMS)-based liquid cells with continuous spacers (Figure 2C) exemplify this configuration.^78^

In open cell configurations, the composition of the solution can be controlled from the outside of the liquid cell, and appropriate models depend on the acting mass transport mechanisms: For diffusion-dominated setups (Figure 2D),^79^ a 1D heterogeneous implementation with open boundary and (typically) radial symmetry (Figure 2H) may be sufficient. Relevant examples comprise the Liquid flow holder by Hummingbird Scientific^80^ as well as recently introduced *diffusion cells.*^79^ For flow-dominated setups (Figure 2E), where convective transport is superimposed to diffusion, 3D geometric models (Figure 2I) become necessary,^48^ although geometric simplifications might apply in specific parameter ranges. Examples include DENSsolutions’ Stream system^81^ and other customized and commercial systems.^67^^,^^77^ Finally, experimental scenarios involving electric potentials/fields tend to increase the geometric complexity of the required model. These scenarios, along with scanning irradiation scenarios, are beyond the scope of this manuscript due to the absence of established benchmarks.

## Results and discussion

### Workflow demonstration with a reference water set

The designed workflow was first tested with the established reference water set from Schneider *et al.*^17^

### Filtering, transfer, and expansion

For that, the *global* reaction database file (see above and STAR Methods section) was loaded in AuRaCh and filtered for the reactions in which 15 species^17^ are involved using the *AuRaCh2COMSOL* script in AuRaCh and manual sparsing. The *reaction network* table obtained contained 73 reactions and was equivalent to the reaction set reported by Schneider and co-workers,^17^ yet it was obtained semi-automatically in a fraction of time. The obtained network was successfully checked for mass and charge balance in AuRaCh.

**Figure 3:**
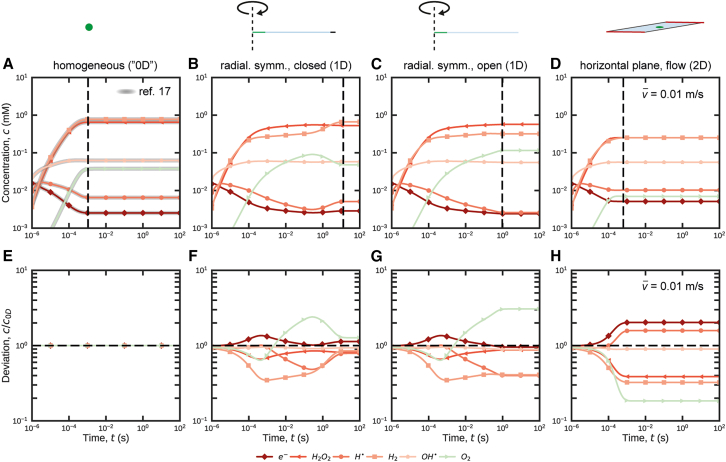
Radiolytic response of an established water set modeled in different realistic irradiation scenarios (A–D) Time-dependent concentration curves of radiolytic species (averaged across the irradiated region) for homogeneous (A), 1D radially symmetric with closed (B) and open (C) boundary conditions considering diffusion as well as 2D/3D geometry considering diffusive and convective transport (D). Relevant model parameters were the electron beam dose rate (Ψ = 7.5⋅10^7^ Gy s^−1^), beam radius (1 μm; in B–D), width of the non-irradiated section (*w*_LC_ = 50 μm, in (B and C), liquid layer thickness (*t* = 150 nm; in (D) and mean flow velocity ($\bar{v}$ = 0.01 m s^−1^; in (D). In (A), the results from Schneider *et al.* (implemented by hand)^34^ are successfully reproduced (*gray curves*).^17^ The temporal evolution of the concentration curves, the relative steady-state concentration values of the radiolytic species and the time required to reach them (10^−3^ s, 10^1^ s, 10^0^ s and 10^−3^ s, respectively, *dashed black lines* in (A–D) strongly depend on the applied boundary conditions. (E–H) Calculated deviation of the concentration curves in (A–D) from the homogeneous reference model by Schneider and co-workers (indicated by *black dashed lines* in (E–H).^17^ Values smaller/larger than 1 correspond to smaller/larger concentration values compared to the reference model, respectively. Note that deviations up to 3*x* are widely observed and occasionally reach ∼10*x* for some byproducts (e.g., O_3_, see Figure S3).

Next, the *Rad-4D* MATLAB script was applied to transfer the AuRaCh output files (*reaction network* and *species* table) and the *local variables* table to COMSOL. In COMSOL, a homogeneous (“0D”) model was generated following established methodology.^17^^,^^34^ Initial and boundary conditions were defined in analogy to the reference model (see also Supporting information section 1C–E),^35^ and a time-dependent solution was (re-)calculated (Figure 3A). The entire process from filtering the *global* database in AuRaCh to obtaining a time-dependent solution in COMSOL can be realized in <1 h, if the *global* reaction database file contains the *reaction network* of interest.

Finally, the network was expanded in space (see STAR Methods section) and solved for the three most relevant LP-EM scenarios of increased geometric and physical complexity (compare Figures 2C–2E).

Figures 3B–3D depicts the temporal evolution of the concentration of the radiolytic species averaged across the irradiated region computed for the three described space-dependent scenarios, namely radially symmetric 1D closed (Figure 3B) and open (Figure 3C) diffusion models and 2D/3D diffusion and flow (Figure 3D) models.

### Validation

The constructed models were validated against previous implementations of the reference water set, where applicable. The homogeneous (“0D”) solution is equivalent to the solution presented by Schneider *et al.* (*gray curves* in Figure 3A), demonstrating the capability of the workflow for flawless construction of chemical reaction networks.^17^ While the concentration evolution for the 1D open model (Figure 3B) is in good agreement with the benchmark results (compare *center curves* for H_2_, e_h_^−^, O_2_, and H^+^ in Figures 3B, 3D, 3G, and 3H reported by Schneider *et al.,*^17^ respectively). With time-dependent implementations of radiolysis models including convection in a 3D geometry are non-existent, our previous 2D stationary models serve as a restricted ref.^34^ On that basis, we performed extensive studies on the flow model dimensions (see Figure S2). We found that the average time-dependent concentration profile of the most accurate 3D implementation is well approximated by a horizontal 2D model (depicted in Figure 3D), rather than a vertical (unless the width of the flow channel is comparable to the width of the irradiated spot), for the range of mean flow velocities ($\bar{v}$ ≲ 10^−2^ m s^−1^) and beam radii (*r >* 100 nm) considered realistic for LP-EM.^48^^,^^79^

### General analysis

The reader is referred to established literature for fundamental chemical aspects of the space-independent reaction network.^17^^,^^74^ Here, we limit the analysis to differences in the evolution of the water reaction network due to the physical and geometrical expansion inside the irradiated region. To facilitate comparison, the concentration of representative species (e_h_^−^, H⋅, OH⋅, O_2_, H_2_, H_2_O_2_)^17^ was averaged across the irradiated region (radius *r* = 1 μm). The data (Figures 3A–3D) is complemented with the deviation of each solution from the homogeneous implementation (in Figure 3A) in Figures 3E–3H.

In heterogeneous (1D) irradiation scenarios (Figures 3B and 3C), an irradiated reaction network (“inside the beam”) is coupled with a non-irradiated one (“outside”) *via* diffusion. On relatively short time scales, the evolution inside the beam region is dominated by the outward diffusion of radiolytic species but independent of the boundary condition (open/closed).^34^ That timescale depends on the lateral expansion of the LC geometry. For an LC width of *w*_LC_ ≈ 50 μm, it is *t* ≤ 10^−1^ s as estimated based on the mean square displacement (*t* = *w*_LC_/8*D*_H+_ ≈ 0.08 s, with *D*_H+_ = 9⋅10^−9^ m^2^ s^−1^ being the diffusion coefficient of the fastest diffusing H^+^ species),^17^^,^^82^ and apparent from the congruent evolution of all curves in Figures 3B and 3C up to this moment in time. In both 1D scenarios, the transient concentrations of primary radiolytic species within the beam region are lower compared to the homogeneous case. However, most secondary species experience an increase, up to 3-fold for O_2_ (at *t* ≈ 10^−1^ s). These trends are attributed to the beam-generated species, which diffuse outwards and consequently lower the self-scavenging capabilities of the network (see Figure S5).^34^

On longer time scales (*t* > 10^−1^ s for *w*_LC_ ≈ 50 μm), the boundary conditions significantly influence the response of the reaction network inside the beam region. The continuity requirements of *open* boundaries lead to the rather fast emergence of a steady state (after *t ≈* 1 s). In contrast, closed boundaries cause the accumulation and back-diffusion of stable radiolytic species (see Figure S5). Consecutively, their (self-) scavenging effect on the network inside the beam region is observed, leading to different steady-state concentrations reached after a certain time delay (*t ≈* 10^1^ s) compared to the open scenario.

Notably, the deviation from the homogeneous case is minor for closed cell configurations, while stronger deviations persist in the open scenario, although still within 1 order of magnitude (see Figures 3G, S3G, and S4). However, in contrast to closed setups, in which the final composition is predetermined through the initial concentration, open scenarios allow the network to be tuned by (dynamically) controlling the solution composition at the inlet.^34^

Additional simulations agree with previous reports on the increase of the steady-state concentration with dose rate,^17^ which is further found to be largely independent of the applied boundary conditions (see Figures S6–S9). Moreover, they suggest a much stronger dependence of the time *t*_ss_ to reach steady-state concentrations (*c*_ss_) on the lateral extension but not the *c*_ss_ itself (see Figures S10–S13).

When high mean flow velocities are applied (here: $\bar{v}$ *=* 10^−2^ m s^−1^), convection becomes the dominant mass transport, leading to a seemingly improved control over the response of the radiolytic reaction network inside the beam region (see Figure 3D). Not only are stationary conditions established after short times (*t ≈* 10^−3^ s), which are comparable to those of the homogeneous (“0D”) case, but the deviations do not exceed 5*x* (Figure 3H) at any time. Yet, low-concentration byproducts such as O_3_ occasionally exceed this limit (see Figure S3H). Notably, and in contrast to the reaction-diffusion scenarios (Figures 3B and 3C), the species for which the stationary average concentrations increase most are the highly reductive hydrated electron (e_h_^−^) and hydrogen radical (H^·^). This is due to the removal of the secondary species (note their deviation being <1 in Figure 3H) and the resulting weakening of the self-scavenging capability of the network.^34^ Moreover, additional simulations confirmed recent observations^45^ that *t*_ss_ decreases with increasing flow rate and decreasing beam radius but is largely independent of the dose rate (see Figure S14–S19).

Comparable to the open diffusion scenario, convection-dominated configurations also allow the reaction network to be tuned by controlling the composition of the inflowing solution; however, the unidirectional flow breaks the radial symmetry of the concentration profiles, such that radiolytic effects may become highly dependent on the location (inside the beam region).^34^ Due to the parabolic flow profile along the vertical axis, i.e., parallel to the beam direction, the effect may significantly vary with the vertical position inside the flow channel, depending on the velocity regime and the beam radius.^34^ The responsiveness to control the reaction network by chemical scavenging strategies (through solution replacement) strongly depends on the details of the channel geometry, both for diffusion- and flow-dominated scenarios.^79^

### Workflow demonstration with the reference gold set

The workflow was also tested for the more complex sparse gold set.^35^

### Filtering, transfer, and expansion

Again, the reaction set was obtained by filtering the *global* database for the reactions where the 22 species were involved. Comparable to the water set, the gold set obtained through filtering comprised more reactions than the reference sparse gold set.^35^ Excess reaction equations were taken out automatically after the manual removal of species from the reaction network table, and the sparsed gold set was successfully checked for mass and charge balance.

**Figure 4:**
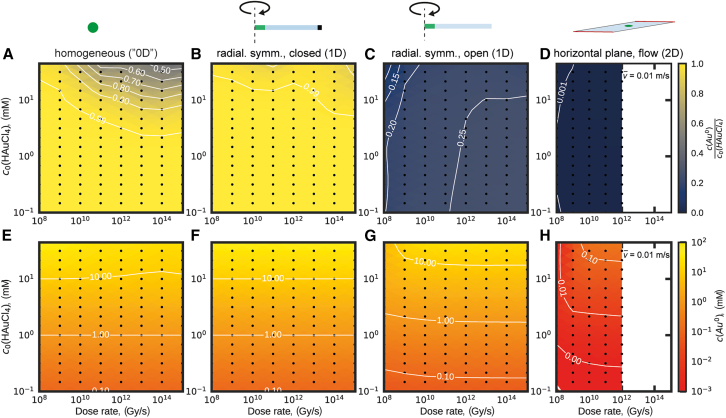
Radiolytic response of an established sparse gold set modeled in different realistic irradiation scenarios (A–D) Colormaps indicating the proportion of reduced gold among all gold species (*c*(Au^0^)/*c*_0_(HAuCl_4_)) averaged across the irradiated region displayed for a range of dose rates and initial gold concentration for homogeneous (“0D”) (A), 1D radially symmetric with closed (B) and open (C) boundary condition considering diffusion as well as 2D geometry considering diffusive and convective transport (D). The electron beam dose rate was 10^8^ Gy s^−1^ < Ψ < 10^15^ Gy s^−1^ and the initial HAuCl_4_ concentration was 10^−1^ mM < *c*_0_(HAuCl_4_) < 5⋅10^1^ mM. Other relevant model parameters were the beam radius (1 μm; in (B–D), extension of the non-irradiated section (*w*_LC_*=* 50 μm, in (B–D) and mean flow velocity ($\bar{v}$ = 0.01 m s^−1^; in (D)). In (A), the results from Fritsch *et al.* are successfully reproduced.^33^ (E–H) Colormaps indicating the absolute concentration of reduced gold (Au^0^) averaged across the irradiated region are displayed for the same parameter range as in (A–D). Dramatic increase in computational cost prevented solving the sparse gold set in the flow scenario (D) at dose rates >10^12^ Gy s^−1^.

Like for the water set, the Rad-4D script was applied to transfer the AuRaCh output files (*reaction network* and *species* table) together with the *local variables* table to COMSOL where a homogeneous (“0D”) model of the sparse gold set was generated. Initial and boundary conditions were replicated from the reference model (see also Supporting information section 1B – E).^35^ The time-dependent solution was (re-)calculated (Figure 4A).

Next, the network was expanded and solved for the same three expanded LP-EM scenarios as above: homogeneous (i.e., “0D”), radially symmetric 1D closed and open diffusion models, and 2D diffusion and flow models. To facilitate accessibility of the data, colormaps of the stationary *c*(Au^0^)/*c*(HAuCl_4_) ratio averaged over the beam-irradiated region in the parameter space formed by the electron flux density and the initial gold concentration (*c*(HAuCl_4_)) were plotted in analogy to our previous^33^ study (Figures 4A–4D) and complemented by colormaps of the averaged *c*(Au^0^) inside the irradiated region across the same parameter space (Figures 4E–4H).

### Validation

**Figure 5:**
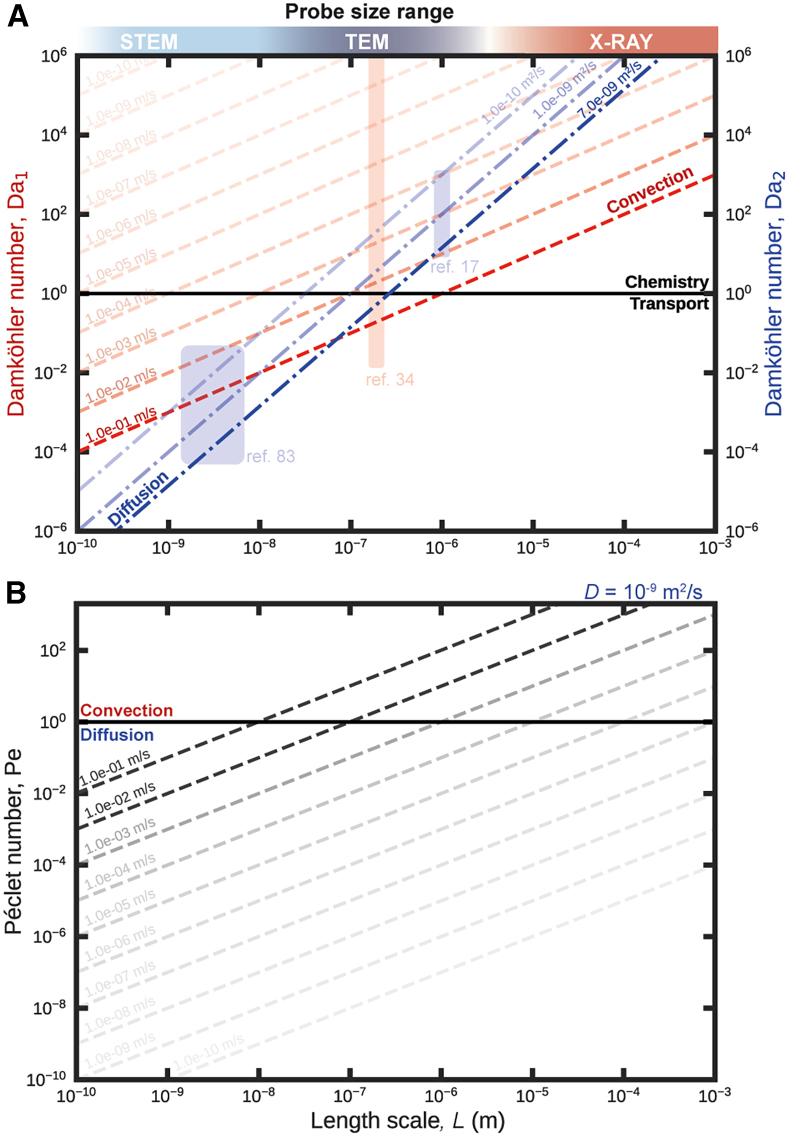
Simplification analysis of radiolysis models based on characteristic numbers (A and B) The Damköhler, *Da*_1_ and *Da*_2_ (A), and the Péclet, *Pe* (B), numbers over a range of length scales (10^−10^ < *L* < 10^−3^ m) relevant for radiation-based sample characterization techniques, i.e., (scanning-) transmission electron microscopy ((S)TEM) –and X-ray-based (X-ray) techniques are depicted. (A) *Da*_1_ and *Da*_2_ are estimated based on Equations 3 and 4, respectively. *Red dashed lines* depict *Da*_1_ for mean flow velocities 10^−10^ m s^−1^ < $\bar{v}$ < 10^−1^ m s^−1^; *blue dash-dotted lines* depict *Da*_2_ for diffusion coefficients 10^−10^ m^2^ s^−1^ < *D* < 7⋅10^−9^ m^2^ s^−1^. In both cases, a characteristic chemical reaction rate *r*_R_ = 10^5^ s^−1^ was assumed. *Red* and *blue rectangles* represent parameter regimes studied by Schneider *et al.*,^17^ Merkens *et al.*,^34^ and Schwarz,^83^ respectively. (B) *Pe* was calculated for 10^−10^ m s^−1^ < $\bar{v}$ < 10^−1^ m s^−1^ and *D* = 10^−9^ m^2^ s^−1^ (*gray dashed lines*). If *Da*_2_ > 1 (*horizontal black line* in (A) and, if applicable, *Da*_1_ > 1, the effect of diffusive, respectively convective, mass transport on a chemical system is negligible. If *Da* < 1, transport phenomena significantly influence chemical reactions. In such case, *Pe* > 1 (horizontal *black line* in (B) implies that convection dominates over diffusion, whereas *Pe* < 1 indicates the opposite*.* Note that the *L* at which *Da*_2_ for *D* = 10^−9^ m^2^ s^−1^ intersects with *Da*_1_ depicted in (A), coincides with the transition of *Pe* from the convection to the diffusion regime in (B).

The constructed models of the sparse gold set were validated based on the implementation of the chemical reaction network, as demonstrated by the perfect match of the data in Figure 4A with previous results (note the different color range compared to Figure 5 reported by Fritsch *et al.*^33^). Geometric and physical expansions were validated via thorough comparison with the complementary implementations of the water set (compare discussion of Figure 3). The flow scenario was implemented in a horizontal 2D geometry following extensive geometric analysis in context of the water set, drastically reducing the computational costs, without significant loss of modeling accuracy for the selected parameters ($\bar{v}$ = 0.01 m s^−1^ and *r* = 1 μm).

### General analysis

As for the water set, the response of the gold reaction network also depends strongly on geometrical and physical aspects. In confined scenarios, i.e., in the homogeneous (“0D”; Figure 4A) and the heterogeneous 1D case with closed boundaries (Figure 4B), reduced gold species accumulate inside the irradiated region. Notably, the initial gold concentration, *c*_0_(HAuCl_4_), above which conversion happens to be incomplete is even increased in the 1D case (*c*(Au^0^)/*c*_0_(HAuCl_4_) > 0.9 for *c*_0_(HAuCl_4_) < 50 mM) as compared to the homogeneous scenario (*c*(Au^0^)/*c*_0_(HAuCl_4_) > 0.9, for *c*_0_(HAuCl_4_) < 10 mM at ≈ 10^13^ Gy s^−1^).

On the contrary, the situation is flipped for scenarios with open boundaries, where diffusion (Figure 4C) or flow (Figure 4D) renews the irradiated reaction solution. In fact, the ratio *c*(Au^0^)/*c*_0_(HAuCl_4_) is reduced with values not exceeding 0.2 for the diffusion- and 0.005 for the convection-governed scenario, respectively. In both scenarios, a higher initial HAuCl_4_ concentration, *c*_0_(HAuCl_4_), is required to achieve Au^0^ concentrations (*c*(Au^0^); Figures 4G and 4H) comparable to that in the homogeneous (“0D”) and 1D closed scenario (Figures 4E and 4F, respectively).

Similarly to the 0D scenario,^33^ the computed results widely agree with previous studies on beam-induced growth kinetics of gold nanocrystals. For instance, Park *et al.* reported a threshold electron flux densities of ≈27 e^−^ Å^−2^ s^−1^ (here equivalent to ≈10^10^ Gy s^−1^ in Figure 4, compare Equation 2), for the crystal growth in a 20 mM HAuCl_4_ solution in a setup comparable to the here simulated 1D open scenario with a beam radius of 1 μm.^36^ In addition, Zhang *et al.* observed diffusion-limited growth, which is typically associated with high monomer concentration, for relatively high electron flux densities (up to 50 e^−^ Å^−2^ s^−1^). In contrast, low HAuCl_4_ concentrations and low electron flux densities led to reaction-limited growth, indicating lower Au^0^ concentration.^84^ Also, our simulations support the authors' qualitative predictions on the effect of flow conditions on shifting the threshold electron flux density for different growth modes.

### Computational costs and accuracy

The discretization of the model geometry (“meshing”) forms the foundation of the applied FE method. With increasing physical complexity, geometrical complexity also increases, which requires more elaborate and computationally intensive meshing strategies. Previously developed^34^ meshing strategies were employed to solve the reference chemical networks (i.e., water and sparse gold;^17^^,^^33^ Figures 3 and 4, respectively) in the respective model geometries (Figures 2F–2I). In each case, mesh refinement studies were conducted to achieve an accuracy threshold of <5%. Under these circumstances, about 10^3^ mesh elements were employed in the 1D models with the finest spatial mesh resolution going down to 0.1 nm in the transition region between the irradiated and non-irradiated model compartments; in the cases of the 2D/3D flow models, the number of elements increased up to ≈6⋅10^4^ and ≈10^6^, respectively. This model growth significantly increased computational costs. While the computation of the time-dependent solution (free logarithmic time stepping between 10^−20^ and 10^2^ s) for one parameter set of the sparse gold set in the homogeneous (“0D”) model took only a few seconds, the computation time required for the 1D scenarios rose to several tens of minutes (≈1.5⋅10^3^ s) and for the 2D flow models to many hours (≈1.5⋅10^4^ s), despite the use of a powerful workstation (Intel Xenon CPU E5-2660 v3) with 512 GB RAM and 10 CPU cores.

The computation time further dramatically increased with dose rate. This was attributed to the increasingly sharp concentration gradient at the edge of the irradiated area caused by the geometry-bound definition of the physical model. Despite considerable efforts, this limitation prevented the computation of steady-state concentrations for the sparse gold set in the flow scenarios at dose rates >10^12^ Gy s^−1^ (see Figures 4D and 4H). The more realistic consideration of the Gaussian beam intensity profile to make this parameter space accessible is beyond the scope of this manuscript (and of subordinate importance, given the emphasis on low-dose techniques in experimental research).

### Additional model simplifications

Thus far, the effectiveness of the proposed workflow in reliably constructing complex models has been demonstrated. However, a dramatic escalation in computational costs associated with implementing high-precision models (both geometrically and physically) was observed. At the same time, only marginal deviations between different scenarios were encountered in certain parameter ranges. These observations urge for the evaluation of additional (physical) model simplification strategies beyond those due to symmetry considerations (see above) and sparsing^35^ of the chemical reaction network.

The Damköhler number, *Da*, is a characteristic dimensionless number that compares the timescales of chemical reactions with that of the mass transport phenomena occurring in a system and is therefore well-suited for such evaluation. Damköhler numbers are defined for convective (*Da*_1_) and diffusive (*Da*_2_) transport according to Equations 3 and 4;^68^

(Equation 3) $${Da}_{1}=\frac{r_{R}}{r_{C}}=r_{R}\left(\frac{L}{\bar{v}\;}\right)\;\text{and}$$

(Equation 4) $${Da}_{2}=\frac{r_{R}}{r_{D}}=r_{R}\left(\frac{L^{2}}{D}\right)\text{,}$$

where $r_{R}$, $r_{C}$ and $r_{D}$ denote the rate of chemical reactions as well as convective and diffusive transport, respectively. The transport rates are conveniently defined as $r_{C}=\bar{v}/L$ and $r_{D}=D/L^{2}$, where $\bar{v}$*, D* and *L* are the mean flow velocity of a solvent, the diffusion coefficient of a solute in that solvent and the characteristic length scale of the system, respectively.^68^ In our previous work, we derived $r_{R}=R_{i}^{\text{cons}}/c_{i}=1/\tau_{i}$ to express the characteristic timescale of a species in a radiolytic reaction network, where $R_{i}^{\text{cons}}$ represents the sum of consumption rates for the species *i*, and $\tau_{i}$ it’s lifetime.^34^

According to Equations 3 and 4, the impact of mass transport on a chemical network decrease when the corresponding Damköhler number increases. In the *transport regime* (*Da* < 1), a diffusive and/or convective mass transport significantly affects chemical reactions, while this effect becomes negligible in the *chemistry regime* (*Da* > 1). Note that *Da*_2_ can be calculated for any scenario in which concentration gradients arise, e.g., due to heterogeneous irradiation, while *Da*_1_ only applies in scenarios that additionally comprise fluid flow. In the latter scenario, the ratio of both Damköhler numbers provides an estimate of the dominant mass transport mechanism – the Péclet number (Equation 5):^68^

(Equation 5) $$Pe=\frac{Da_{2}}{Da_{1}}=\frac{r_{c}}{r_{D}}=\frac{vL}{D}\text{,}$$

with *Pe >* 1 and *Pe* < 1 indicating that convection, respectively diffusion, dominates.

Figures 5A and 5B depicts *Da*_1_ and *Da*_2_ as well as *Pe* for 10^−10^ m < *L <* 10^−3^ m, covering the length scales characteristic for STEM (spur dimension: ≈10^−9^ m; pixel-to-pixel-distance: ≈10^−8^ m, field of view: 10^−7^ – 10^−5^ m), TEM (field of view: 10^−7^ – 10^−5^ m) and X-ray-based (probe size: >10^−6^ m) techniques. Transport rates were estimated within experimentally relevant parameter ranges: 10^−10^ m s^−1^ < $\bar{v}$ *<* 10^−1^ m s^−1^ (*Da*_1_, *red dashed lines*) and 10^−10^ m^2^ s^−1^ < *D <* 7⋅10^−9^ m^2^ s^−1^ (*Da*_2_, *blue dash-dotted lines*), respectively.^34^^,^^48^ The characteristic reaction rate *r*_R_ of a radiolytic network was estimated from the steady-state concentration (*c*_i,ss_) of the homogeneous solution of the water set (Figure 3A), and the corresponding consumption rates $R_{i}^{\text{cons}}$. With *c*_i,ss_ of unstable^25^ species not exceeding *c*_OH⋅,ss_ ≈ 0.1 mM and $R_{i}^{\text{cons}}$ ≤ 10^4^ mM s^−1^, we obtained *r*_R_ ≥ 10^5^ s^−1^ (Ψ = 7.5⋅10^7^ Gy s^−1^), which is in line with previous estimations.^34^^,^^35^

Different parameter regimes in which physical (and associated geometric) model simplifications apply, in addition to those mentioned under symmetry considerations (see above), are apparent from Figure 5. For large characteristic length scales (*L* > 10^−7^ m), a radiolysis network is in the chemistry regime, where a homogeneous implementation that neglects all mass transport mechanisms is expected to provide a reasonably accurate estimation of in-beam reaction kinetics unless extremely high flow velocities are applied ($\bar{v}$ ≫ 10^−1^ m s^−1^; see Figure 5A). This is particularly relevant for X-ray-based techniques with probe sizes typically ≫10^−6^ m but was also described for (low-magnification) TEM imaging (*blue rectangle* in Figure 5A; *Da*_2_ > 10^1^ for a beam radius of 1 μm).^17^^,^^85^

For intermediate characteristic length scales (10^−8^ m < *L* < 10^−6^ m), which correspond to (high-resolution) TEM (and STEM) applications, both Damköhler numbers eventually become comparable and ≈1, depending on the applied $\bar{v}$ and governing *D* (Figures 5A and 5B). This indicates a complex interplay of chemistry, diffusion, and convection, which requires a thorough evaluation of the experimental parameters, and, eventually, the comprehensive modeling of all aspects. These considerations are well reflected in our recent works on the effect of flow on radiolysis in TEM irradiation scenarios using comprehensive reaction-diffusion-convection models^34^^,^^45^: While for $\bar{v}$ < 10^−4^ m s^−1^, the water radiolysis network was found in the chemistry regime with only negligible contribution of (diffusive) mass transport (*Da*_2_ ≈ 10^1^ for *D* = 10^−9^ m^2^ s^−1^; compare *red rectangle* in Figure 5A with Figure 3 of Merkens et al^34^), complex non-linearities arise for 10^−4^ m s^−1^ ≤ $\bar{v}$ < 10^−1^ m s^−1^ due to the enhanced interplay reflected in *Da*_2_ ≥ *Da*_1_ ≈ 1.

Finally, the network reaches the convection-dominated transport regime for $\bar{v}$ > 10^−1^ m s^−1^ (*Da*_1_ < 10^−1^ < *Da*_2_).^34^ The sensitivity of the system toward applied mass transport for intermediate *L* is reflected in the transition of the Péclet number from a convection- into a diffusion-controlled regime (see Figure 5B). Moreover, for systems with Damköhler numbers close to 1, the dose rate Ψ becomes a crucial parameter for driving a radiolysis network in either the transport or chemistry regime due to its direct proportionality to the steady-state concentrations of radiolytic species, and therefore reaction rates.^17^ Similar arguments may apply for the composition of the irradiated solution/network.

For small characteristic length scales (*L* < 10^−8^ m), the estimated Damköhler numbers indicate that systems are in the transport regime, with diffusion being the dominant mass transport (*Da*_1_ > *Da*_2_ ≪1), unless extremely high velocities ($\bar{v}$ > 10^−1^ m s^−1^) are reached. Therefore, modeling STEM-specific phenomena, such as spur-spur and pixel-pixel interactions, should account for diffusive transport,^86^ but only requires convection in extreme scenarios. This necessity is reflected in previous approaches addressing spur evolution in pulse radiation studies (*blue square*).^83^ Note that the Damköhler numbers calculated for the transient regime of TEM (sub-(milli)-second timescale; compare Figures 3A–3D) and for most STEM scenarios are likely to be overestimated. The overestimation results from the lower concentrations expected in these scenarios, which reduces the chemical reaction rate *r*_R_, thereby decreasing both *Da*_1_ and *Da*_2_ proportionally. This further underlines the importance of considering mass transport phenomena, particularly diffusion, in these scenarios (compare TEM results in the transient time regime depicted in Figures 3B and 3C). However, the characteristic length scales associated with the different mass transport phenomena are not necessarily equivalent.

### Limitations of the study

The reported analysis was focused on in-beam effects due to the availability of reference models for validation. However, the introduced workflow facilitates the investigation of out-of-beam phenomena, which will be addressed in follow-up work.

The sparse gold set served as a computationally cheap reference model. Even though the network sparsening was previously validated in a homogeneous scenario, it remains pending for the geometrically and physically more complex scenarios described here.

The spatially homogeneous dose rate employed reflects a simplification for many use cases. Studies on the beam quality, comprising for instance nature, energy spread, and collimation of the probe beam are therefore expected to further increase the accuracy of the computed concentration profiles.

Also note that the reported workflow for transferring complex chemical networks into expanded models is general. It allows all terms of Equation 1 to be (de-)activated independently, including the first-right-hand term describing beam-induced generation. By setting all *G-*values to 0, it can be adapted to fields beyond radiation chemistry, such as chemical reactor kinetics, microfluidic synthesis or biochemistry.

### Conclusion

A comprehensive workflow for realistic radiation chemistry modeling in radiation-based sample characterization methods was introduced. The workflow streamlines the assembly of reaction networks from a (global) database, their automated verification as well as transfer and expansion in FE computational environments. The versatility of the modeling workflow was demonstrated by solving two radiation chemistry reaction networks in various experimentally relevant scenarios. Beyond homogeneous (“0D”) reaction kinetic models, water and sparse gold reaction sets were implemented in radially symmetric 1D reaction-diffusion models with open and closed boundary conditions and 2D/3D reaction-diffusion-flow models. Validation against literature models and comprehensive analysis elaborates the scope of chemically, geometrically, and physically accurate implementations at different complexity levels to achieve experimental relevance. Models were discussed with respect to accuracy and computational costs, and the potential for further simplifications was evaluated based on characteristic numbers for experimentally relevant parameters (regimes). The presented workflow will strengthen the role of computational modeling in establishing a more correlative methodology in radiation-based sample characterization tools such as LP-EM and X-ray-based techniques. We propose a unified global reaction database to propel the efforts of the research community and boost model integrity.

## Resource availability

### Lead contact

Further information and requests for resources should be directed to and will be fulfilled by the lead contact, Andreas Hutzler (a.hutzler@fz-juelich.de).

### Materials availability

This study did not generate new unique reagents.

### Data and code availability

• The AuRaCh routine relies on open-source software and is available on Github (https://github.com/BirkFritsch/Radiolysis-simulations) together with a global database file (reaction library).
• The Rad-4D script together with exemplary database files and a step-by-step description of the workflow are available on Github (https://github.com/GDSalvo/Radiolysis-simulations-with-Rad4D-).
• Any additional information required to reanalyze the data reported in this paper is available from the lead contact upon request.

## Acknowledgments

This work was supported by the 10.13039/501100003086Basque Government in the scope of Elkartek 2023 (KK-2023/00001), the 10.13039/501100019124Diputación Foral de Gipuzkoa (RED2019) and the Spanish MINECO under the Maria de Maeztu Units of Excellence Program (MDM-2016-0618). A.K., B.F., and A.H. gratefully acknowledge financial support via the project StacIE (funding No. 03HY103H) within the H_2_Giga flagship project of the German Federal Ministry of Education and Research (10.13039/501100002347BMBF). The authors thank Marek Grzelczak of the Center for Material Research in San Sebastian for fruitful discussions on radiation chemistry and resources.

## Author contributions

Conceptualization, G.D., S.M., B.F., A.H., and A.C.; workflow implementation, G.D., S.M., and B.F.; AuRaCh expansion, G.D., S.M., B.F., A.K., and A.H.; 1D, 2D, 3D COMSOL simulations, G.D. and S.M.; data curation and analysis, G.D., S.M., B.F., and A.K., writing—original draft, G.D., S.M., and B.F.; writing—review and editing, B.D., S.M., B.F., A.K., P.M., A.H., and A.C.; funding acquisition, A.H. and A.C.; resources, A.H. and A.C.; supervision, S.M., P.M., A.H., and A.C.

## Declaration of interests

The authors declare no competing interests.

## Declaration of generative AI and AI-assisted technologies in the writing process

During the preparation of this work, the authors used large language models, such as Deepl.com, to refine the language of the manuscript. After its usage, the authors reviewed and edited the content as needed and take full responsibility for the content of the publication.

## STAR★Methods

### Key resources table

**Table:** 

REAGENT OR RESOURCE	SOURCE	IDENTIFIER
**Software and algorithms**		

AuRaCh – automated radiation chemistry tool	Birk Fritsch Helmholtz Institute Erlangen-Nürnberg for Renewable Energy	https://github.com/BirkFritsch/Radiolysis-simulations
Rad-4D	Giuseppe De Salvo CIC nanoGUNE	https://github.com/GDSalvo/Radiolysis-simulations-with-Rad4D-
COMSOL Multiphysics	COMSOL, Inc.	https://www.comsol.com/
MATLAB	The MathWorks, Inc.	https://www.mathworks.com/products/matlab.html

### Method details

#### Automated radiation chemistry modeling tool

AuRaCh is an established Python-based tool for simulating (radiation) reaction kinetic networks.^33^ It operates with plaintext files of the chemical reaction set as input, automatically assembles the corresponding matrix of coupled differential equations and computes the time-dependent and spatially homogeneous (“0D”) solution, *i.e.,* concentration of all species. In this work, the capabilities of AuRaCh were extended to *i*) generate reaction network input files by filtering a *global* chemical reaction library (illustrated in Figure S1) for user-defined input commands, *ii*) generate output files that allow the transfer of the chemical model into frameworks for finite element modeling (*e.g.,* COMSOL Multiphysics)^87^ of geometric and physical expansions (see below).

#### AuRaCh-to-COMSOL model transfer

Rad-4D, introduced here, is a customized Livelink-MATLAB script that generates COMSOL Multiphysics files (.mph) for time-dependent simulations of (radiation) chemistry networks in physically and geometrically complex (*i.e.,* 4D) scenarios. Rad-4D is configured to work with validated output files from AuRaCh. The workflow provides users with a one-click solution for their individual use case, requiring little experience in scientific programming. Relevant *G*-values and diffusion coefficients are reproduced in Tables S1 and S2.

#### Finite element modeling framework

Physically and geometrically complex models were implemented in COMSOL Multiphysics software (version 5.4) by extending the validated chemical (*reaction engineering*) models imported from AuRaCh with Rad-4D (see above). Two previously reported reaction networks, *i.e.,* water^17^ and (sparse) gold^33^^,^^35^ set, were investigated. The implementations aimed to replicate common experimental scenarios for LP-EM: The radiolytic generation of species was ascribed to a restricted region with a radius of 1 μm, applying dose rates of 10^8^ Gy s^−1^ < Ψ < 10^15^ Gy s^−1^. Reaction-diffusion models were built with radial 1D symmetry (model width: 50 μm), while the reaction-diffusion-flow models were constructed in 2D and 3D geometry, exploring mirror symmetry.^34^ The initial conditions (*i.e.,* the composition of the irradiated solution) were reproduced from the reference models,^17^^,^^35^ correcting for mass and charge balance. In reaction-diffusion models, the boundaries were defined as closed and open, respectively. For the water set, the initial volume ($c_{0,i,2D}$) and inlet ($c_{0,i,\text{inlet}}$) concentration, both in the 1D open and 2D/3D flow models, were equal to $c_{0,i}$ = 0 for all species *i* with exception of $c_{0,H+}=c_{0,\text{OH}-}=10^{-7}$ M and $c_{0,H20}=55.56$ M. For the sparse gold set, the HAuCl_4_ was additionally assumed to fully dissociate, *i.e.,* $c_{0,\text{AuCl}4-}=c_{\text{HAuCl}4}$, taking values between 10^−1^ and 5⋅10^1^ mM. The effect of the acid on the *p*H, respectively *p*OH prior to irradiation, was considered as $c_{0,H+}=$ $10^{-7}M+c_{\text{HAuCl}4}$ and *p*H + *p*OH = 14. For the sparse gold set the initial and inlet concentration of O_2_ was $c_{0,02}=0.255\;M\text{.}$ In reaction-diffusion-convection models, the inlet boundaries were assigned a mean flow velocity ($\bar{v}$ = 0.01 m s^−1^) and the volume and outlet pressures were defined 0. The flow velocity at the walls (*v*_wall_ = 0; no-slip condition) and mass transport through the channel walls was zero for all species: $\nabla c_{i}\cdot {\hat{n}}_{wall}=0$. The downstream edge was described as outlet for mass transport with Equation 6 and for flow using pressure boundary conditions (Equation 7).

(Equation 6) $$-n\cdot D_{i}\nabla c_{i}=0$$

(Equation 7) $$[-pI+\mu (\nabla v+{\left(\nabla v\right)}^{T}]n=-\hat{p_{0}}n,\text{with}\;\hat{p_{0}}\leq p_{0}$$

For the open models, the composition of the entering solution was defined as identical to the initial composition. For more information on implementing diffusion, refer to previous literature.^34^ Convective transport was implemented through the laminar flow module.^34^^,^^48^ Laminar flow and diffusion were coupled in the Multiphysics node. The required spatial and temporal resolution of the mesh and solver applied were determined in a mesh refinement study following previously introduced strategies.^34^ Finite element simulations were conducted on an Intel Xenon CPU E5-2660 v3 workstation (10 cores, 512 GB RAM).

#### Model validation

Implemented models were validated following previously established methodology.^34^^,^^48^^,^^79^ Of particular importance here was the validation of 2D and 3D flow models. The average time-dependent concentration profile of the most accurate 3D implementation is well approximated by both a horizontal as well as a vertical 2D model (see Figure S2) for the parameters tested in this manuscript, i.e., mean flow velocities ($\bar{v}$ ≲ 10^−2^ m s^−1^) and beam radii (*r* = 1 μm). In a previous study where smaller beam sizes were applied, the horizontal and vertical 2D models diverge due to the different diffusion symmetries (compare Figure 5).^48^^,^^79^

### Quantification and statistical analysis

There are no quantification or statistical analyses to include in this study.
