# Supplementary material for: A workflow for modeling radiolysis in chemically, physically, and geometrically complex scenarios
Source: iScience. 2025 Apr 8;28(5):112374. doi: 10.1016/j.isci.2025.112374 (PMC12063123; doi:10.1016/j.isci.2025.112374)
Supplement: Document S1. Figures S1–S19 and Tables S1 and S2 [file mmc1.pdf]

iScience, Volume *28*

Supplemental information

A workflow for modeling radiolysis in chemically, physically, and geometrically complex scenarios

Giuseppe De Salvo, Stefan Merkens, Andreas Körner, Birk Fritsch, Paolo Malgaretti, Andreas Hutzler, and Andrey Chuvilin

1. Supplemental figures

a. Chemical reactions

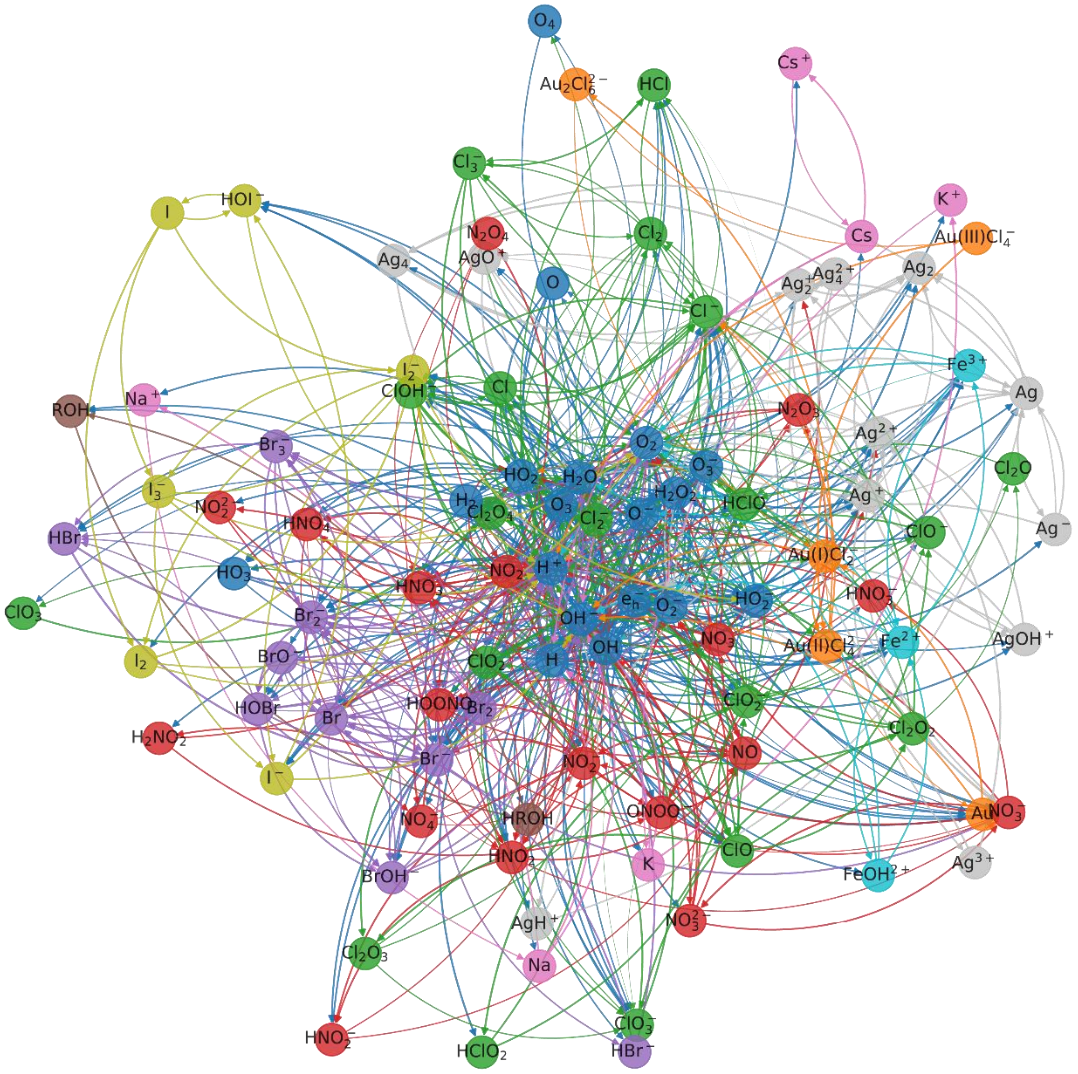

Figure S1. Network representation of a *global* database, as composed in this study. In this study, the underlying database file was filtered (and manually sparsed) to obtain the water set and the sparse gold set introduced by Schneider and co-workers and in our previous study, respectively. Related to the *Automated radiation chemistry modeling tool* section.

b. Model Validation: Water set – 2D *vs* 3D flow

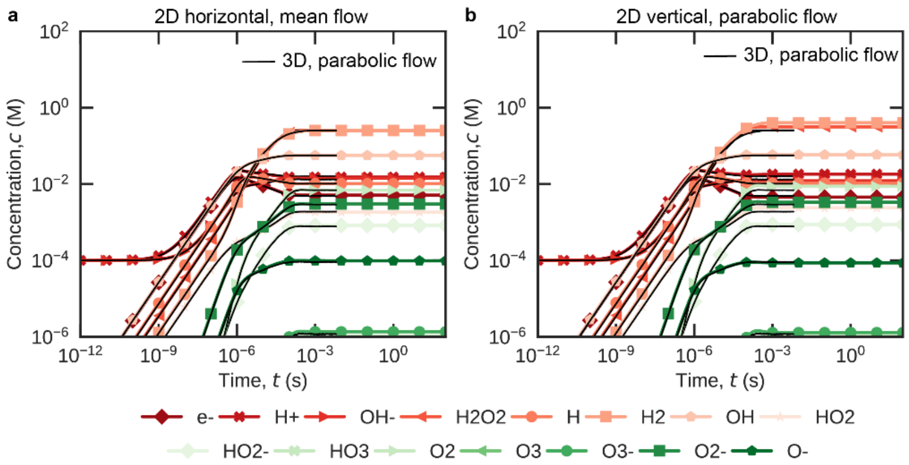

Figure S2. Geometric simplification of flow models (water set). Comparison of geometrically simplified 2D flow models, compared to the most accurate geometric representation, *i.e.,* 3D geometry with parabolic flow profile. For the selected parameter set, *i.e.,* beam radius r = 1 µm, dose rate ψ = 7.5·10^{7} Gy s^{-1}, mean flow velocities $\bar{v}$ = 0.01 m s^{-1}, both simplified geometric models, *i.e.*, 2D horizontal (A) and 2D vertical (B), the concentration, c, averaged in the irradiated region is equal to the accurate solution of the 3D model (*black lines*). The height t of the 3D model was 150 nm. Note that for different parameter set, *e.g.,* smaller r,[1] deviations from the accurate 3D model are expected. For this manuscript the 2D (horizontal) model is a valid model simplification. Related to the *Validation* section of the reference water set.

c. **Water Set**

i. **All species**

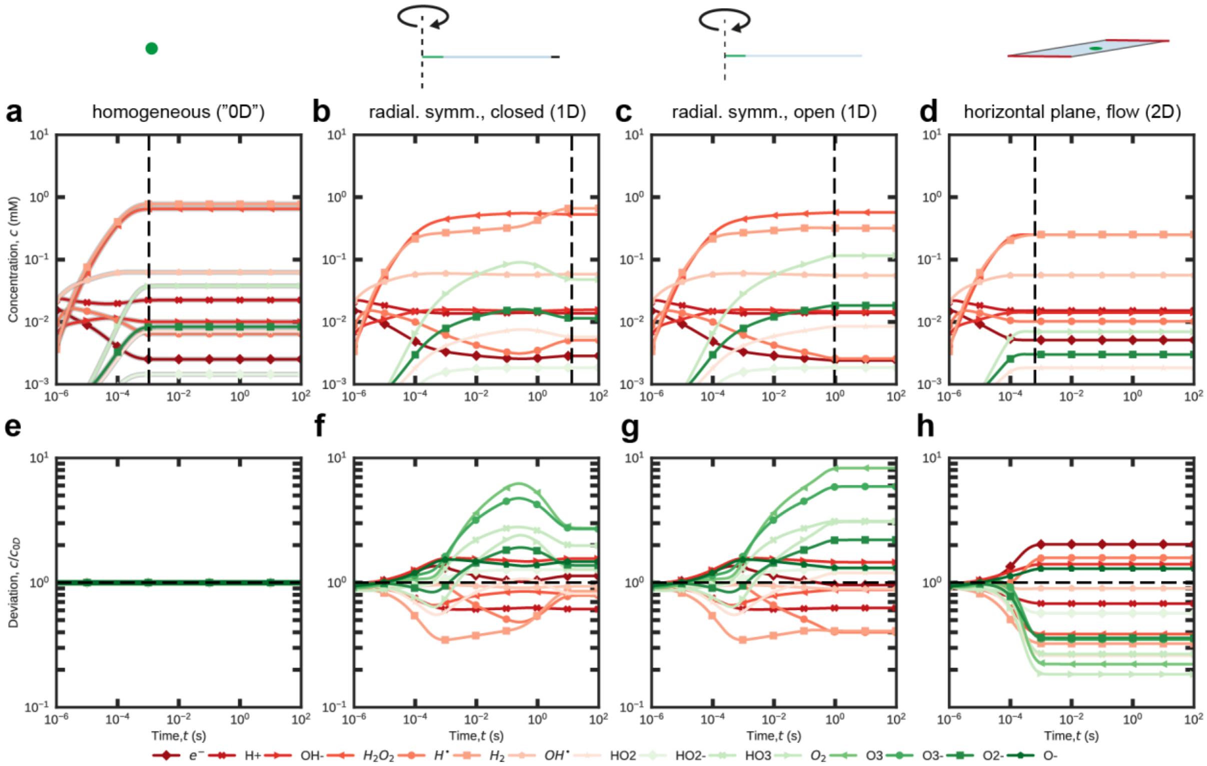

Figure S3. Radiolytic response of an established water set modeled in different realistic irradiation scenarios. Complete reaction set of the data depicted in **Figure 3**.

ii. **Radiolytic Acidity**

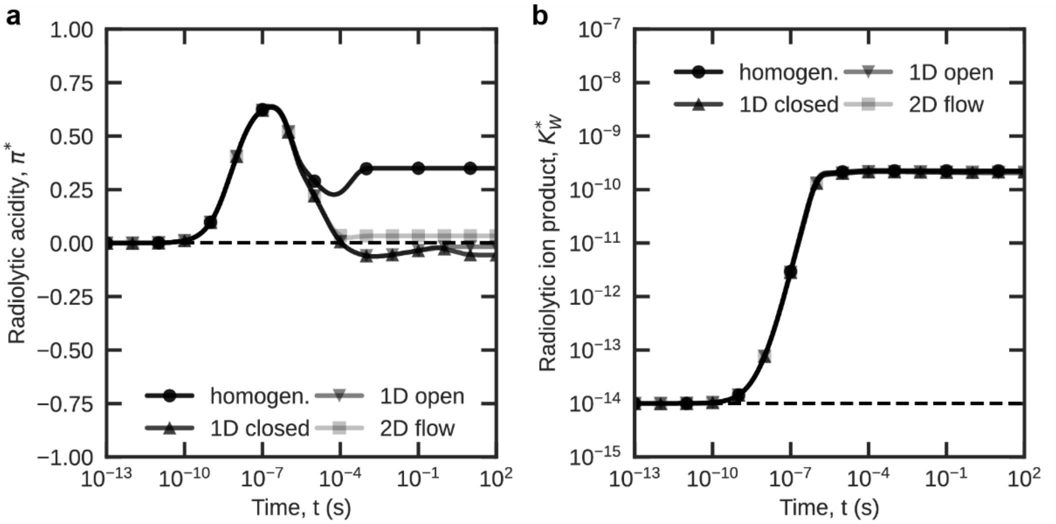

Figure S4. Temporal evolution of the radiolytic acidity (A) and radiolytic ion product (B) calculated from the water set based on definition in Fritsch *et al.*[2] for the model implementations reported in **Figure 3**.

iii. Boundary effect in diffusion-reaction model

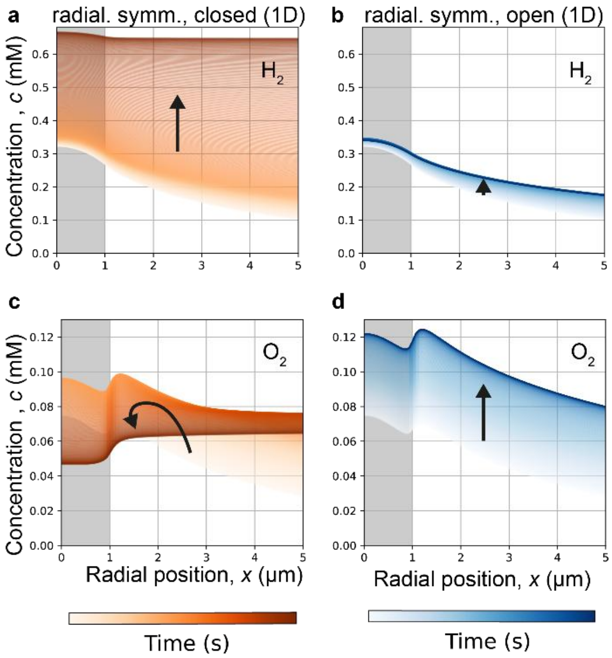

Figure S5. Saturation & back diffusion in radially symmetric irradiation scenarios (1D model). (A – D) Concentration of the stable gaseous species (hydrogen, (A and B), and oxygen, (C and D) in the radially symmetric 1D geometry with closed (A and C) and open (B and D) boundaries, respectively. Width of liquid cell w_{LC} = 50 µm; beam radius (*grey*) r = 1 µm in both models. Related to the *General analysis* section of the reference water set.

iv. Effect of dose rate (radial. symm., 1D closed)

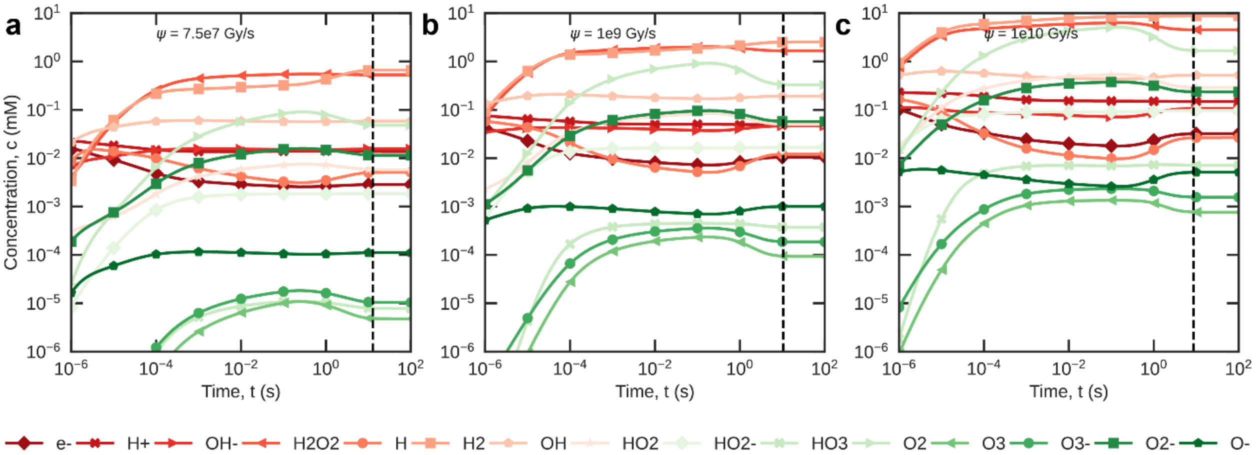

Figure S6. Effect of dose rate Ψ on the response of an established water set under inhomogeneous irradiation and closed boundary conditions. Time-dependent concentration curves of radiolytic species (averaged across the irradiated region), for $\Psi = 7.5 \cdot 10^7$ Gy s^{-1} (A), 10^9 Gy s^{-1} (B) and 10^{10} Gy s^{-1} (C). Model width and beam radius were w_{LC} = 50 µm and r = 1 µm. Data in **a** is equivalent to **Figure 3B**. Concentration at inlet were defined in section 1E.

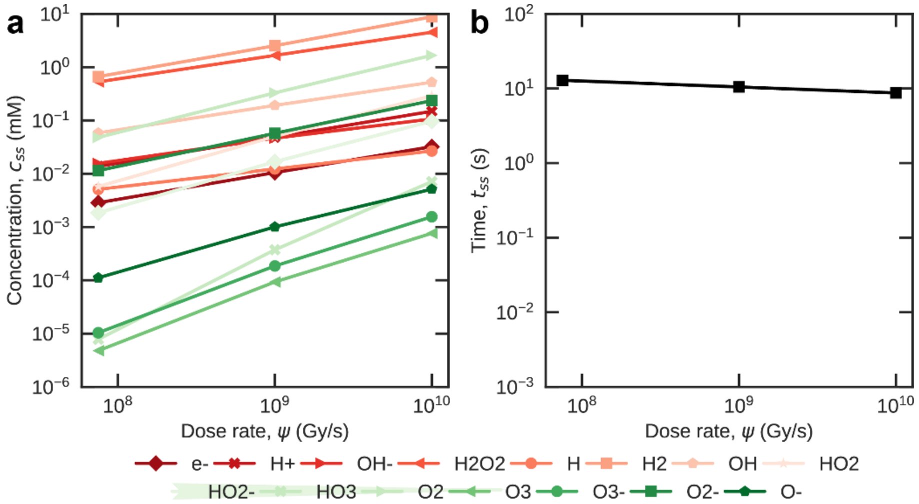

Figure S7. Effect of dose rate Ψ on the steady-state concentration c_{ss} (A) and the time t_{ss} to reach c_{ss} (B) of an established water set under inhomogeneous irradiation and closed boundary conditions. Data is a subset of **Figure S6**.

v. Effect of dose rate radial. symm., 1D open)

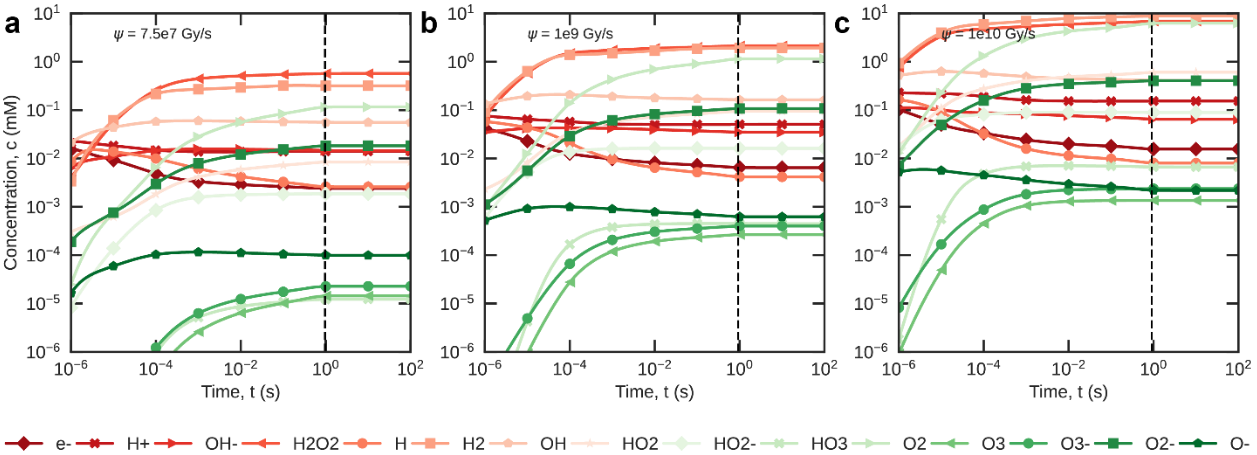

Figure S8. Effect of dose rate Ψ on the response of an established water set under inhomogeneous irradiation and open boundary conditions. Time-dependent concentration curves of radiolytic species (averaged across the irradiated region), for $\Psi = 7.5 \cdot 10^7$ Gy s^{-1} (**a**), 10^9 Gy s^{-1} (B) and 10^{10} Gy s^{-1} (C). Model width and beam radius were w_{LC} = 50 µm and r = 1 µm. Data in (A) is equivalent to **Figure 3B**. Concentration at inlet were defined in section 1E.

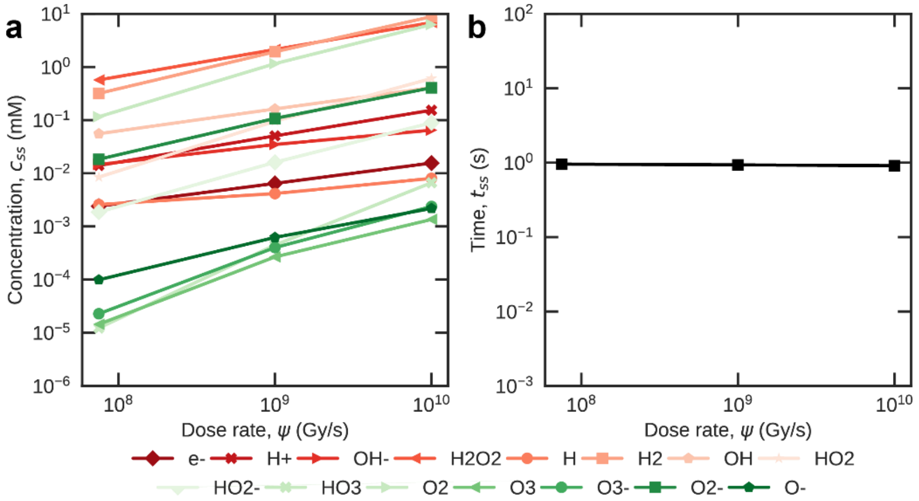

Figure S9. Effect of dose rate Ψ on the steady-state concentration c_{ss} (A) and the time t_{ss} to reach c_{ss} (B) of an established water set under inhomogeneous irradiation and open boundary conditions. Data is a subset of **Figure S8**.

vi. Effect of model extension (radial. symm., 1D closed)

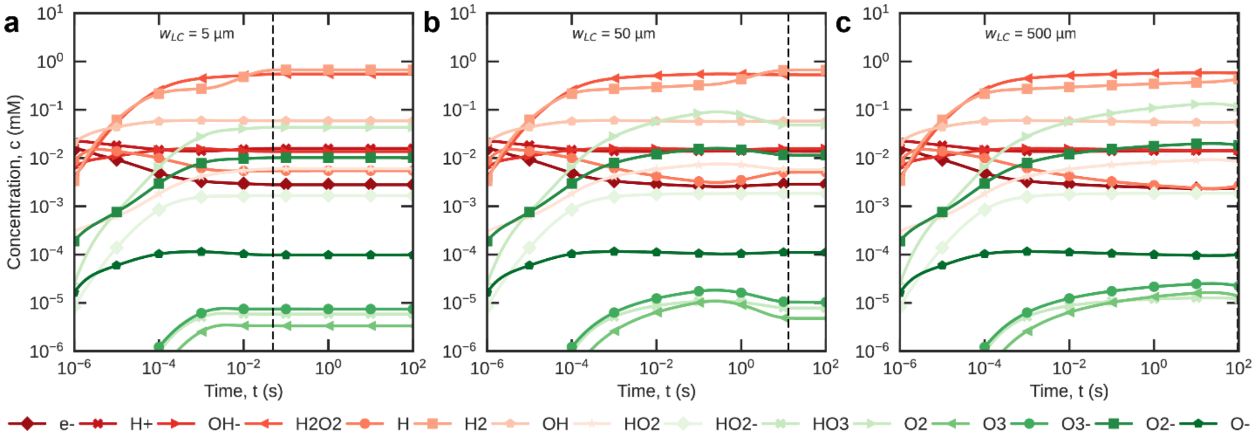

Figure S10: Effect of model extension w_{LC} on the response of an established water set under inhomogeneous irradiation and closed boundary conditions. Time-dependent concentration curves of radiolytic species (averaged across the irradiated region), for w_{LC} = 5 µm (A), 50 µm (B) and 500 µm (C). Dose rate and beam radius were $\Psi = 7.5 \cdot 10^7$ Gy s^{-1} and r = 1 µm. Data in (B) is equivalent to **Figure 3B**. Concentration at inlet were defined in section 1E.

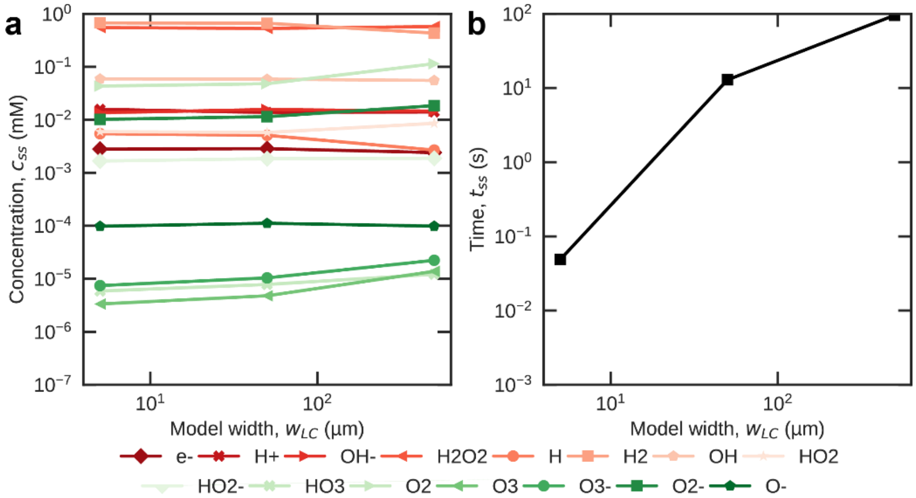

Figure S11: Effect of model extension w_{LC} on the steady-state concentration c_{ss} (A) and the time t_{ss} to reach c_{ss} (B) of an established water set under inhomogeneous irradiation and closed boundary conditions. Data is a subset of **Figure S10**.

vii. Effect of model extension w_{LC} (radial. symm., 1D open)

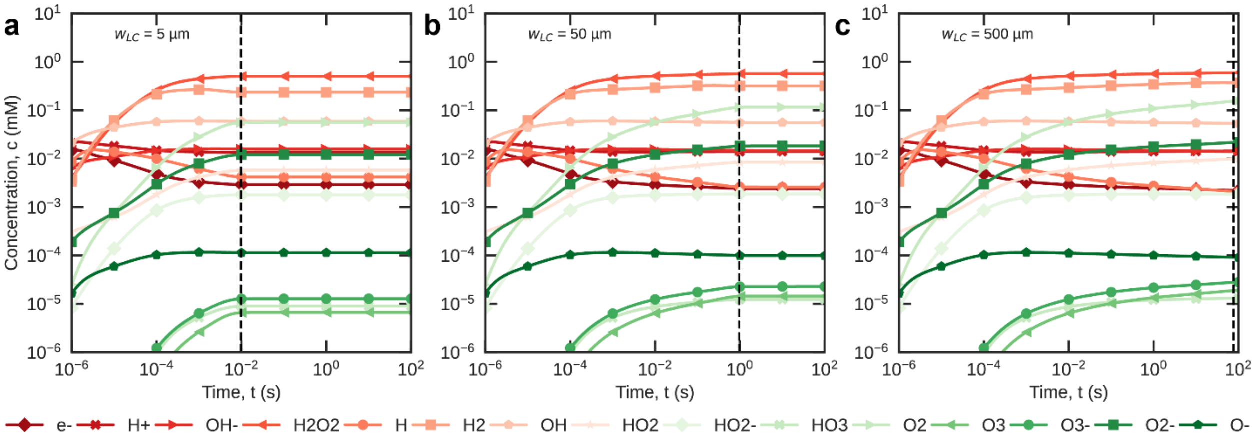

Figure S12: Effect of model extension w_{LC} on the response of an established water set under inhomogeneous irradiation and open boundary conditions. Time-dependent concentration curves of radiolytic species (averaged across the irradiated region), for w_{LC} = 5 µm (A), 50 µm (B) and 500 µm (C). Dose rate and beam radius were $\Psi = 7.5 \cdot 10^{7}$ Gy s^{-1} and r = 1 µm. Data in (B) is equivalent to **Figure 3B**. Concentration at inlet were defined in section 1E.

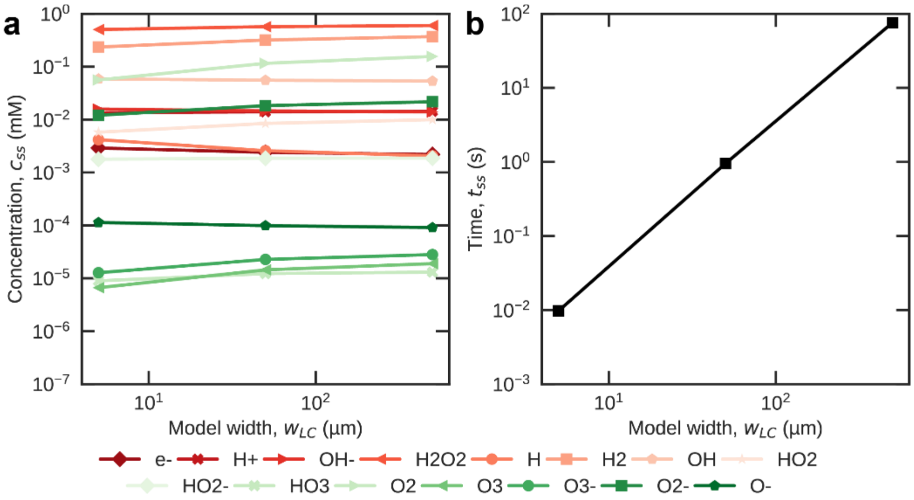

Figure S13: Effect of model extension w_{LC} on the steady-state concentration c_{ss} (A) and the time t_{ss} to reach c_{ss} (B) of an established water set under inhomogeneous irradiation and open boundary conditions. Data is a subset of **Figure S12**.

viii. Effect of dose rate (2D flow)

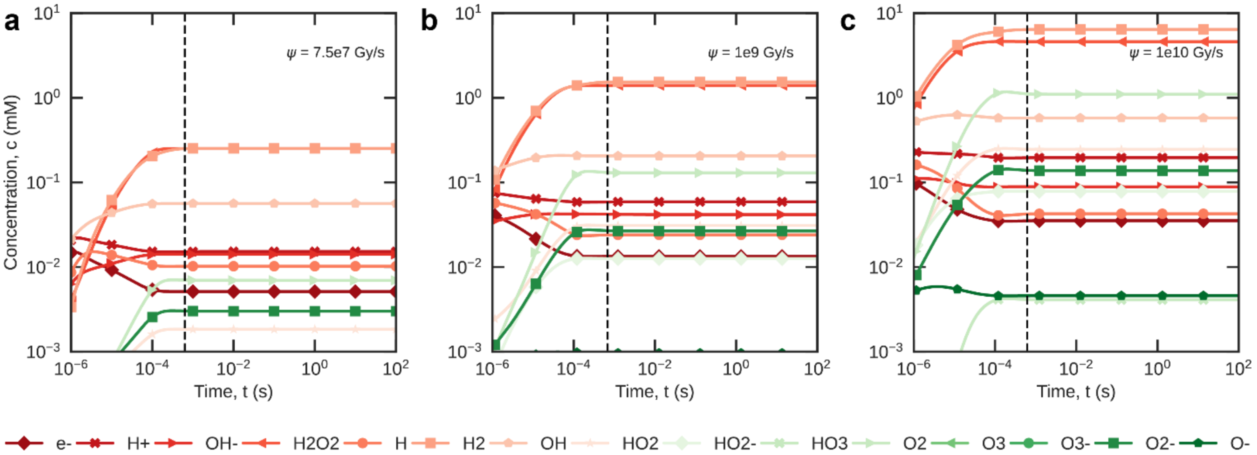

Figure S14: Effect of dose rate Ψ on the response of an established water set under flow conditions. Time-dependent concentration curves of radiolytic species (averaged across the irradiated region), for $\Psi = 7.5 \cdot 10^7$ Gy s^{-1} (A), 10^9 Gy s^{-1} (B) and 10^{10} Gy s^{-1} (C). Mean flow velocity and beam radius were $\bar{v}$ = 0.01 m s^{-1} and r = 1 µm. Data in (A) is equivalent to **Figure 3D**.

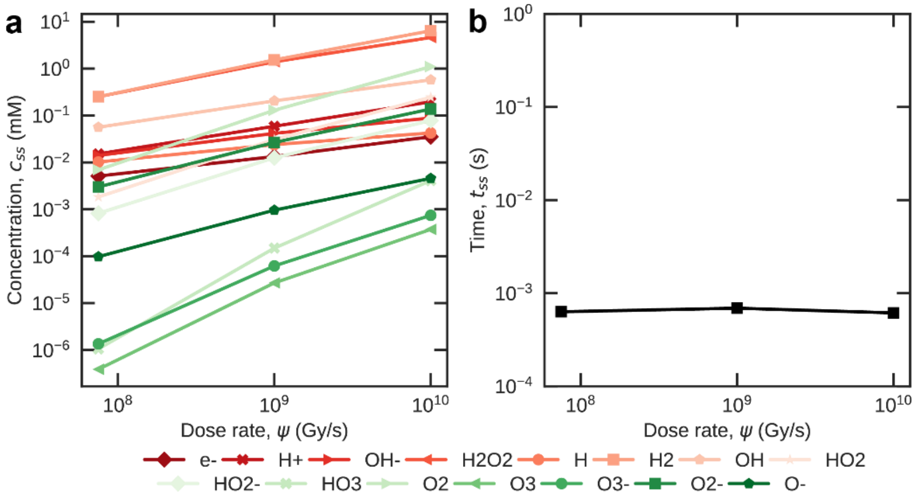

Figure S15: Effect of dose rate Ψ on the steady-state concentration c_{ss} (A) and the time t_{ss} to reach c_{ss} (A) of an established water set under flow conditions. Data is a subset of **Figure S14**.

ix. Effect of beam radius (2D flow)

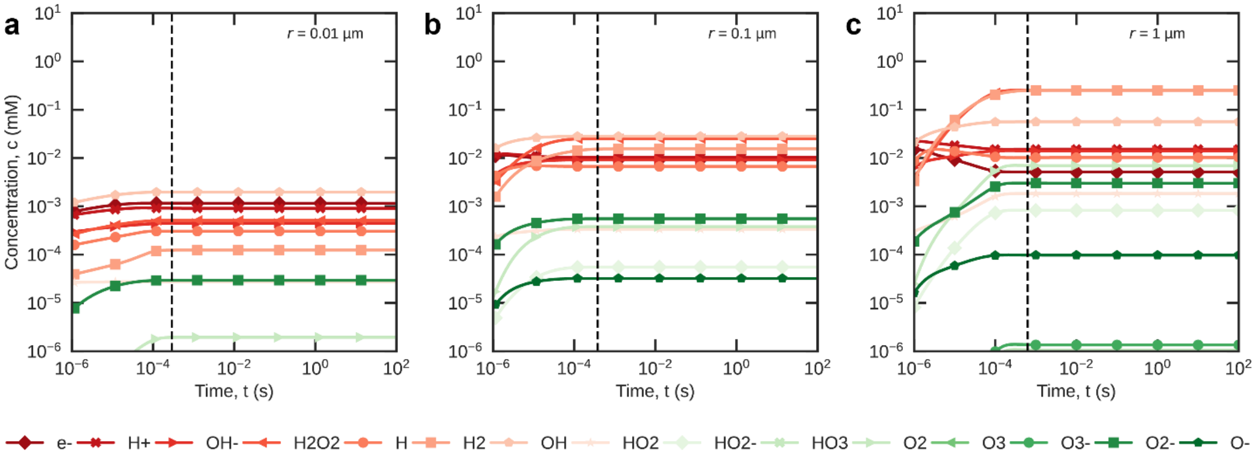

Figure S16: Effect of beam radius *r* on the response of an established water set under flow conditions. Time-dependent concentration curves of radiolytic species (averaged across the irradiated region), for *r* = 0.01 µm (A), 0.1 µm (B) and 1 µm (C). Mean flow velocity and dose rate were $\bar{v}$ = 0.01 m s^{-1} and Ψ = 7.5·10^{7} Gy s^{-1}. Data in (C) is equivalent to **Figure 3D**.

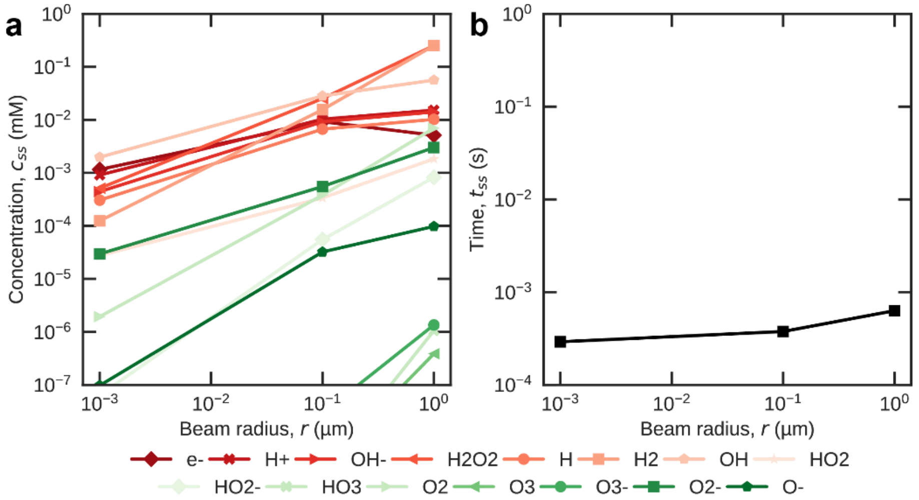

Figure S17: Effect of beam radius *r* on the steady-state concentration c_{ss} (A) and the time t_{ss} to reach c_{ss} (B) of an established water set under flow conditions. Data is a subset of **Figure S16**.

x. Effect of flow velocity (2D flow)

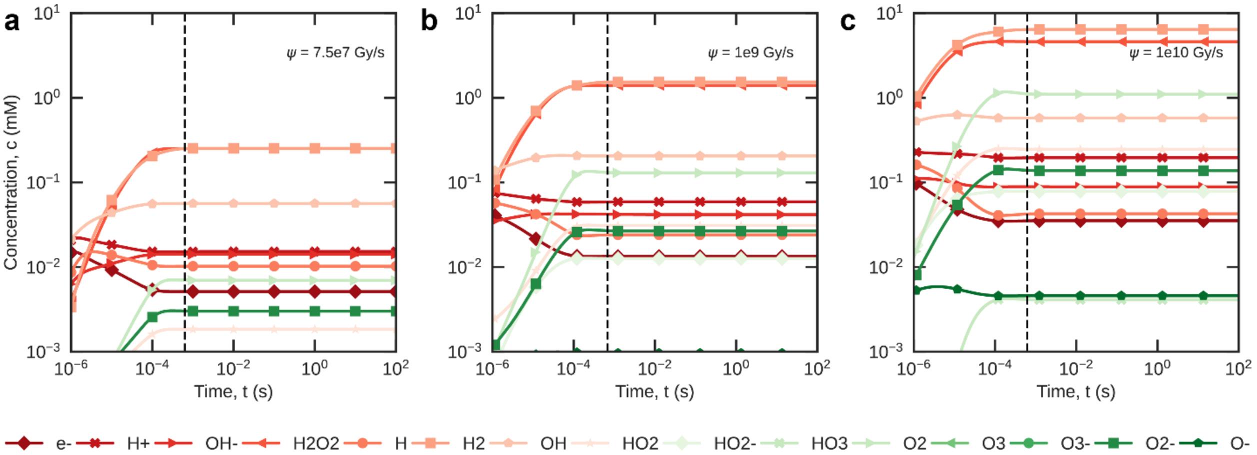

Figure S18: Effect of mean flow velocity $\bar{v}$ on the response of an established water set under flow conditions. Time-dependent concentration curves of radiolytic species (averaged across the irradiated region), for $\bar{v}$ = 0.0001 m s^{-1} (A), 0.001 m s^{-1} (B) and 0.01 m s^{-1} (C). Beam radius and dose rate were r = 1 µm and $\Psi = 7.5 \cdot 10^{7}$ Gy s^{-1}. Data in (C) is equivalent to **Figure 3D**.

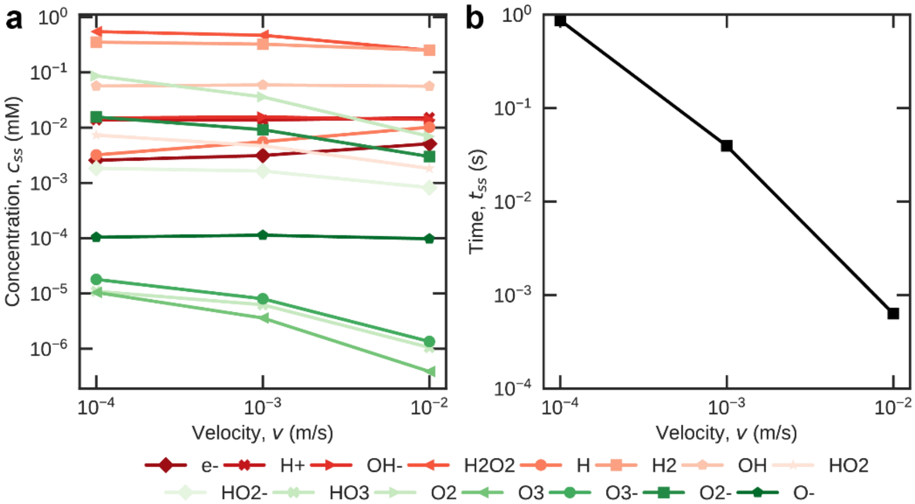

Figure S19: Effect of beam radius r on the steady-state concentration c_{ss} (A) and the time t_{ss} to reach c_{ss} (B) of an established water set under flow conditions. Data is a subset of **Figure S18**.

2. Supplemental tables

a. Radiolytic generation

Table S1: Relevant *G* values.

Species	G values		Species	G values	
	(#/100 eV)	(10^{-7} mol J^{-1})		(#/100 eV)	(10^{-7} mol J^{-1})
**Au**	0	0	**$H^{\cdot}$**	1 [3]	1.04
**$AuCl_4^-$**	0	0	**H^+**	4.42 [3]	4.58
**$Au_2Cl_6^{2-}$**	0	0	**H_2**	0.17 [3]	0.18
**$AuCl_2^-$**	0	0	**H_2O_2**	0.47 [3]	0.49
**$AuCl_4^{2-}$**	0	0	**HO_2**	0.08 [3]	0.08
**Cl**	0	0	**HO_2^-**	0	0
**Cl^-**	0	0	**O_2**	0	0
**Cl_2^-**	0	0	**O_2^-**	0	0
**$ClOH^-$**	0	0	**$OH^{\cdot}$**	3.63 [3]	3.76
**HCl**	0	0	**OH^-**	0.95 [3]	0.98
**H_2O**	-5.68	-5.89	**e_h^-**	3.47 [3]	3.60

* Note the alternative annotation in SI units, together with modifications of the radiolytic generation term in *Eq.* 1, were reported in previous work.[4]

b. Diffusion

Table S2: *Relevant diffusion coefficients.*

Species	Diffusion Coefficient *D* (· 10^{-9} m^2 s^{-1})	Species	Diffusion Coefficient *D* (· 10^{-9} m^2 s^{-1})
**Au**	1.9 [5]	**$H^{\cdot}$**	7[3]
**$AuCl_4^-$**	1.9 [6]	**H^+**	9 [3]
**$Au_2Cl_6^{2-}$**	$D_{\mathrm{AuCl4-}}/4$	**H_2**	4.5 [3]
**$AuCl_2^-$**	2.4 [6]	**H_2O_2**	1.4 [3]
**$AuCl_4^{2-}$**	$D_{\mathrm{AuCl4-}}/2$	**HO_2**	2 [3]
**Cl**	3 [5]	**HO_2^-**	1.4 [3]
**Cl^-**	2 [7]	**O_2**	2.1 [3]
**Cl_2^-**	$D_{\mathrm{Cl-}}/2$	**O_2^-**	2.1 [3]
**$ClOH^-$**	1.1 [8]	**$OH^{\cdot}$**	2.8 [3]
**HCl**	3.2 [9]	**OH^-**	5 [3]
		**e_h^-**	4.5 [3]

***** The diffusion coefficients of $Au_2Cl_6^{2-}$, $AuCl_4^{2-}$ and Cl_2^- were estimated based on the 1/*q* and 1/*r* dependence of *D.*[10]

3. Supplemental references

[S1] Merkens, S., De Salvo, G. & Chuvilin, A. The Effect of Flow on Radiolysis in Liquid Phase-TEM flow cells. *Nano Express* **3**, 045006 (2023).

[S2] Fritsch, B. *et al*. Tailoring the Acidity of Liquid Media with Ionizing Radiation: Rethinking the Acid-Base Correlation beyond pH. *Journal of Physical Chemistry Letters* **14**, 4644–4651 (2023).

[S3] Schneider, N. M. *et al*. Electron-Water interactions and implications for liquid cell electron microscopy. *J. Phys. Chem. C* **118**, 22373–22382 (2014).

[S4] Sun, J. *et al*. Discovery of Molecular Intermediates and Nonclassical Nanoparticle Formation Mechanisms by Liquid Phase Electron Microscopy and Reaction Throughput Analysis. *Small Struct* **5**, 2400146 (2024).

[S5] Kuss, J., Holzmann, J. & Ludwig, R. An elemental mercury diffusion coefficient for natural waters determined by molecular dynamics simulation. *Environ Sci Technol* **43**, 3183–3186 (2009).

[S6] Nakazato, Y., Taniguchi, K., Ono, S., Eitoku, T. & Katayama, K. Formation dynamics of gold nanoparticles in poly(vinylpyrrolidone) and other protective agent solutions. *Physical Chemistry Chemical Physics* **11**, 10064–10072 (2009).

[S7] aquion. Table of Diffusion Coefficients. *https://www.aqion.de/site/diffusion-coefficients* (2024).

[S8] Chao, M. S. The Diffusion Coefficients of Hypochlorite, Hypochlorous Acid, and Chlorine in Aqueous Media by Chronopotentiometry. *J. Electrochem. Soc.* **2**, 1172–1174 (1968).

[S9] Harpst, J. A., Holt, E. & Lyons, P. A. Diffusion in Dilute Hydrochloric Acid-Water Solutions. *J Phys Chem* **69**, 2333–2335 (1965).

[S10] Crank, J. The Mathematics of Diffusion. (1975).
